# Solution structure of the type I polyketide synthase Pks13 from *Mycobacterium tuberculosis*

**DOI:** 10.1186/s12915-022-01337-9

**Published:** 2022-06-21

**Authors:** Cécile Bon, Stéphanie Cabantous, Sylviane Julien, Valérie Guillet, Christian Chalut, Julie Rima, Yoann Brison, Wladimir Malaga, Angelique Sanchez-Dafun, Sabine Gavalda, Annaïk Quémard, Julien Marcoux, Geoffrey S. Waldo, Christophe Guilhot, Lionel Mourey

**Affiliations:** 1 Institut de Pharmacologie et de Biologie Structurale, IPBS, Université de Toulouse, CNRS, UPS, Toulouse, France; 2 Los Alamos National Laboratory, Bioscience Division B-N2, Los Alamos, NM 87545 USA; 3 Present address: Centre de Recherche en Cancérologie de Toulouse (CRCT), Inserm, Université de Toulouse, CNRS, UPS, Toulouse, France; 4 Present address: Toulouse White Biotechnology, 31400 Toulouse, France; 5 Present address: Carbios, Biopole Clermont Limagne, 63360 Saint-Beauzire, France

**Keywords:** Mycolic acids, Polyketide synthases, Small-angle X-ray scattering, 3D structure

## Abstract

**Background:**

Type I polyketide synthases (PKSs) are multifunctional enzymes responsible for the biosynthesis of a group of diverse natural compounds with biotechnological and pharmaceutical interest called polyketides. The diversity of polyketides is impressive despite the limited set of catalytic domains used by PKSs for biosynthesis, leading to considerable interest in deciphering their structure‐function relationships, which is challenging due to high intrinsic flexibility. Among nineteen polyketide synthases encoded by the genome of *Mycobacterium tuberculosis*, Pks13 is the condensase required for the final condensation step of two long acyl chains in the biosynthetic pathway of mycolic acids, essential components of the cell envelope of *Corynebacterineae* species. It has been validated as a promising druggable target and knowledge of its structure is essential to speed up drug discovery to fight against tuberculosis.

**Results:**

We report here a quasi-atomic model of Pks13 obtained using small-angle X-ray scattering of the entire protein and various molecular subspecies combined with known high-resolution structures of Pks13 domains or structural homologues. As a comparison, the low-resolution structures of two other mycobacterial polyketide synthases, Mas and PpsA from *Mycobacterium bovis* BCG, are also presented. This study highlights a monomeric and elongated state of the enzyme with the apo- and holo-forms being identical at the resolution probed. Catalytic domains are segregated into two parts, which correspond to the condensation reaction per se and to the release of the product, a pivot for the enzyme flexibility being at the interface. The two acyl carrier protein domains are found at opposite sides of the ketosynthase domain and display distinct characteristics in terms of flexibility.

**Conclusions:**

The Pks13 model reported here provides the first structural information on the molecular mechanism of this complex enzyme and opens up new perspectives to develop inhibitors that target the interactions with its enzymatic partners or between catalytic domains within Pks13 itself.

**Supplementary Information:**

The online version contains supplementary material available at 10.1186/s12915-022-01337-9.

## Background

Type I polyketide synthases (PKSs) are multifunctional, large enzymes [1] responsible for the biosynthesis of a wide array of natural compounds, the so-called polyketides [2], which exhibit very interesting and widely used pharmacological properties. They are also involved in important biological processes of various bacteria such as cell wall biogenesis and/or pathogenicity [3]. Type I PKSs conduct decarboxylative Claisen condensation [4] between an aliphatic chain to be elongated and an extender unit, usually malonyl- and methylmalonyl-Coenzyme A (CoA). Having a common evolutionary ancestor with type I fatty acid synthases (FASs), type I PKSs have retained analogous catalytic domain composition. Acyl carrier protein (ACP), acyltransferase (AT) and ketosynthase (KS) are the three mandatory catalytic domains involved in the condensing steps. ACP tethers the growing intermediate, thioesterified to a phosphopantetheine (Ppant) arm covalently attached to a serine residue. AT selects the precursor and/or extender unit. KS catalyses the condensation reaction. Following elongation, the ACP-bound intermediate may be sequentially reduced by ketoreductase (KR), dehydrated by dehydratase (DH) and further reduced by enoylreductase (ER) domains. Downstream modifications may eventually follow or occur concomitantly through a methyltransferase (MT) domain, and the product is then released from ACP, and potentially cyclized, by a thioesterase (TE) or a malonyl/palmitoyl transferase (MPT) domain. In addition to the variability in domain composition, the organization may differ from one type I PKS to another. Indeed, chain elongation and reduction may be conducted iteratively, as in FASs, or may be conducted through various modules on an assembly line, each module containing a set of catalytic domains that will be used once. Such PKSs are called modular, and there is no counterpart in FASs. FASs and PKSs also exist as class II, in which all the catalytic domains required for the catalytic cycle correspond to discrete enzymes [5], and class III, in which a single domain is used to conduct a limited set of chain elongations followed by cyclization [6]. Here and unless otherwise stated, the terms PKSs and FASs will be used for type I PKSs and FASs.

Deciphering the structure-function relationships of PKSs would bring valuable information in order to understand and rationally impact some biological processes and for the bioengineering of new polyketides [7]. Despite this strong interest, PKS structures are difficult to obtain, undoubtedly due to their inherent flexibility [8]. The first structures of such megasynthases were those of yeast and mammalian FASs (mFAS) obtained by X-ray crystallography [9–14]. Later, structures of *Mycobacterium smegmatis* and other fungal FAS enzymes have been solved using cryo-electron microscopy [15–17]. As expected from biochemical data [18], mFAS forms X-shaped dimers whose interface involves the KS, ER and DH domains and which define two lateral catalytic chambers with a clear structural segregation. Unexpectedly, most linker regions were found to be folded, except the small stretches between the condensing and modifying parts that define the central pivot, a fact that explains the remarkable flexibility of the entire enzyme around this central connection [19]. Strikingly, yeast and mycobacterial FASs display a very distinct heterododecameric barrel-shaped architecture where a large amount of the residues corresponds to non-catalytic regions and composes rigid scaffolding elements, with few flexible linkers. An arrangement similar to that in mFAS has been observed for several structures of excised KS-AT didomains [20–24] or AT domains flanked with their pre- and post-linkers [25, 26]. Thus, it was believed that one could extrapolate the arrangement of PKSs and FASs from the mFAS structure, with the help of structural information obtained for mono- or multi-domains, or sequence-related type II monofunctional enzymes. Since then, structural information on several PKSs have been published [20, 27–33] and it became clear that they are much more diverse in structure than previously anticipated, in line with their particularly high product diversity [1, 34–38]. Depending on organisms, FASs may be organized as homodimers, hexamers or heterododecamers [11] whereas PKSs have always been characterized as dimers, although the way they dimerize may be distinct from that of FASs. In addition to their diversity of oligomerization, FASs and PKSs display various architectures, inducing highly distinctive catalytic chambers. More generally, the finely tuned complex arrangement of catalytic domains in PKSs and FASs is either constrained by an extensive scaffolding matrix, where catalytic domains interact via non-enzymatic structured linkers, or relies on direct domain interactions for 3D assembly.

**Fig. 1:**
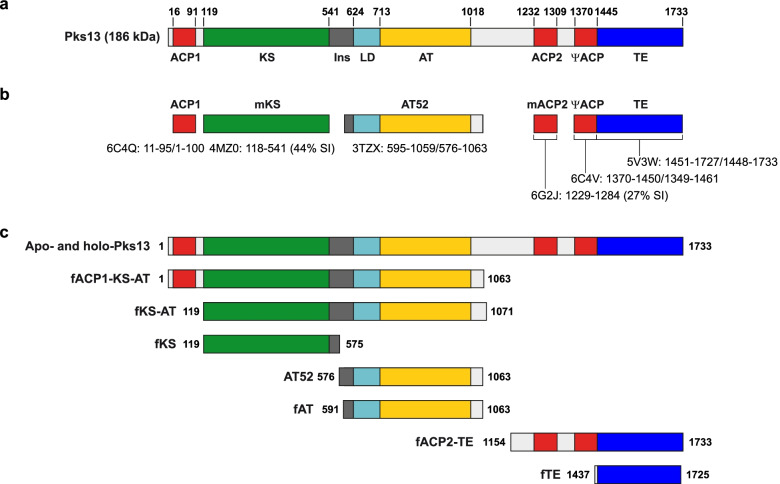
Sequence-scaled linear domain organization of Pks13. **a** Overall view. **b** Structural coverage. PDB codes and residues resolved in the structures/boundaries of the crystallized constructs are indicated. For mKS and mACP2, which were modelled based on homology, percentages of sequence identity (SI) are given. **c** The different molecular entities produced within the current study. Colour code of the domains is as follows: ACP, red; KS, green; AT, yellow; TE, blue; LD, cyan. Interdomain linkers are in grey and the *Mtb* Pks13 insertion (Ins) is in dark grey

The work presented here focuses on Pks13 of *Mycobacterium tuberculosis* (186 kDa, 1733 residues), the condensase involved in the final condensation step leading to the formation of mycolic acids (see Additional file 1: Fig. S1 for details) [39, 40], essential and specific components of the cell envelopes of genera from *Corynebacteriales* order [41, 42]. Pks13 has been validated as a drug target in the fight against tuberculosis [43–46]. Sequence analysis revealed that the enzyme is composed of five catalytic domains: ACP1, KS, AT, ACP2 and TE (Fig. 1a). It has been shown that ACP1 and ACP2 undergo post-translational modification resulting in holo-forms [42, 47]. Pks13 may also be phosphorylated, although no details have been provided [48]. The AT domain is responsible for transferring the C22-C26 α-chain from the corresponding acyl-CoA to the free thiol of the Ppant arm onto ACP2. Holo-ACP1 is involved in loading the C40-C70 meromycolic chain (previously activated under an acyl-AMP form) and its transfer to the KS domain (Additional file 1: Fig. S1). The KS domain then catalyses the decarboxylative condensation between the meromycolic chain and the α-chain to form a α-alkyl β-ketoacyl product. Finally the TE domain catalyses the release of the product on polyols, preferably trehalose [40]. Pks13 is particular in the sense that it is not a modular enzyme but nevertheless performs only one elongation cycle on very long fatty acyl substrates. Furthermore, the meromycolic chain needs to be activated by the fatty acyl-AMP ligase FadD32 [42]. Moreover, FadD32/ACP1 interaction is required for transferring the meromycoloyl chain from AMP onto Pks13. Finally, the product released is transferred onto polyols. Compared to other PKSs, Pks13 possesses a high amount of interdomain linkers, some of which are especially long (i.e. ~200 residues). Two other mycobacterial PKSs, Mas (224 kDa, 2111 residues) and PpsA (199 kDa, 1876 residues) from *M. bovis* BCG, have been studied. These enzymes are involved in the biosynthesis of phthiocerol dimycocerosates (DIMs) and the related phenolic glycolipids (PGLs), major lipid virulence factors [49–51]. Mas (KS-AT-DH-ER-KR-ACP) is an iterative enzyme catalysing the formation of the C24-C30 methyl-branched mycocerosic acids. PpsA (ACP-KS-AT-DH-KR-ACP) is encoded within the *ppsABCDE* modular PKS gene cluster and participates to the formation of the C32-C34 phthiocerol chain.

Using small-angle X-ray scattering (SAXS), we show that Pks13 has an equivalent structure in both apo- and holo-forms at the resolution probed. Unexpectedly, Pks13 is monomeric, though pushed towards dimerization following the loading of a C16-CoA substrate analogue onto the AT domain. The dimer displays a surprisingly open and globally symmetric architecture. Two structural regions were characterized within a monomer, corresponding to ACP1-KS-AT and ACP2-TE. Strikingly, these two regions undergo minor intrinsic flexibility whereas the entire enzyme displays a relatively high flexibility involving a pivot region between the AT and ACP2 domains. We also propose a hybrid model combining our low-resolution structure with high-resolution structures of excised Pks13 domains and structural homologues and compare Pks13 structure to those of Mas and PpsA enzymes.

## Results

### Bioinformatic analysis and strategy used for the structural study

The five catalytic domains of Pks13 as predicted from sequence analysis correspond to residues 16-91 (ACP1), 119-541 (KS), 713-1018 (AT), 1232-1309 (ACP2) and 1445-1733 (TE) (Fig. 1a). This is globally in line with resolved sub-structures: ACP1 from residue 11 to 95 (PDB entry code 6C4Q, unpublished) and 15 to 93 (6D8J, unpublished), AT from residue 713 to 1036 in fragment AT52 (3TZX, [25]) and TE from residue 1451 to 1727 (5V3W, [43]) (Fig. 1b). A fragment, which seems to correspond to a cryptic ACP domain (ψACP), has also been solved (6C4V, residues 1370‑1450, unpublished). This highlights the presence of non-catalytic linker regions containing up to 200 residues. It is noteworthy that the Pks13 KS-to-AT linker (residues 625-712) forms a compact domain in the structure of AT52 [25] and in related structures from FASs and PKSs, where it is often referred to as LD for linker domain. Compared to other PKSs and FASs, Pks13 contains a long insertion (residues 542–624) between KS and LD for which only residues 595–624, forming an α-helix, could be resolved in the AT52 structure. Thus, structural information was lacking for KS and ACP2 and for a large amount of linker regions: ACP1-to-KS, 23 residues; KS-to-LD, 53 residues; AT-to-ACP2: 172 residues; and ACP2-to-ψACP: 60 residues (Fig. 1b).

**Fig. 2:**
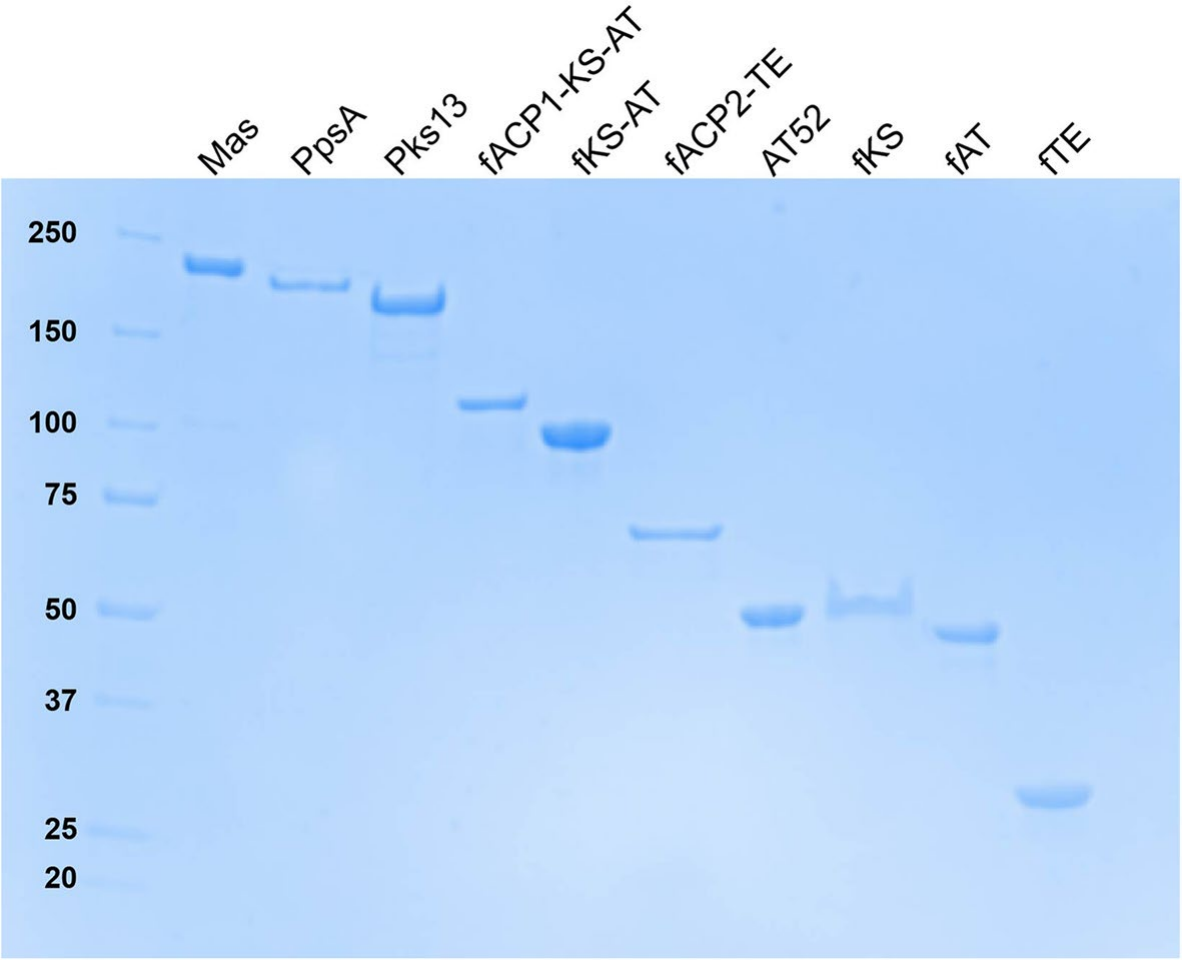
SDS-PAGE of purified Mas, PpsA and Pks13 full-length proteins and Pks13 fragments. Molecular weight ladder is on the first lane

Our strategy for unravelling the Pks13 architecture was based on a hybrid approach where low-resolution structures obtained by SAXS were combined with known crystal structures for Pks13 fragments and structural homologues. Pks13 fragments used for SAXS were designed based on bioinformatic analysis or produced either by limited proteolysis of the purified full-length protein or by domain trapping (see the ‘Methods’ section for more details). Twelve macromolecular entities were used in the current study. As most entities were in the apo-form, the single entity with phosphopantetheine arms has been specifically termed as holo-Pks13. We studied five full-length enzymes, Pks13 and holo-Pks13, Pks13(S1533A) (a mutant needed to avoid artefactual loading of the C16-CoA substrate analogue on the thioesterase domain [42]), Mas and PpsA, and seven Pks13 fragments with MW between 33 and 116 kDa: fACP1-KS-AT, fKS-AT, fKS, fAT, AT52, fACP2-TE and fTE (Fig. 1c). Three other fragments, fACP1-KS, fAT-ACP2 and fAT-ACP2-TE, have been challenged but discarded in the course of this study due to aggregation propensity (see the ‘Methods’ section for details). All corresponding proteins were overproduced from soluble fractions of recombinant *Escherichia coli* strains and their purification protocol determined in standard physicochemical conditions (Fig. 2). Pks13 fragments allowed covering most of the entire Pks13 sequence (Fig. 1c and Table 1). We checked both by native mass spectrometry and bottom-up proteomics that we were analysing non-truncated proteins. Native MS (Additional file 1: Fig. S2) showed that Pks13 has a mass corresponding to a monomer of 188.8 ± 0.05 kDa, 188.0 kDa being the theoretical mass. In the bottom-up study, 90% sequence coverage starting from residue 2 to the end of the C-terminal His_6_-tag has been obtained for Pks13(S1533A), which undoubtedly confirms that we challenged a full-length protein (Additional file 1: Fig. S3). Moreover, as Pks13 surprisingly appeared to be monomeric in the condition probed (both by SAXS and native mass spectrometry), we challenged Pks13(S1533A) following incubation with a C16-CoA substrate analogue in order to test the potential effect on dimerization. The expected covalent loading of the 16-carbon-long chain on the AT catalytic serine (step 3’, Additional file 1: Fig. S1) was validated using bottom-up proteomics (Additional file 1: Fig. S4), and indeed, a tendency to dimerize was then observed.

**Table 1:** Pks13, Mas and PpsA constructs and their global and hydrodynamic properties

Protein ^a^	Pks13 (Holo-Pks13)	Pks13(S1533A) monomer (C16-Pks13(S1533A) dimer)	fACP1‑KS‑AT	fKS‑AT	fKS	fAT	AT52	fACP2‑TE	fTE	Mas monomer (Dimer)	PpsA
Sequence boundaries	1–1733	1–1733	1–1063	119–1071	119–575	591–1046	576–1063	1154–1733	1437–1725	1–2111	1–1876
MW_th_ (kDa), Number of residues	188.0, 1746	188.0, 1746 (376.0, 3492)	115.8, 1084	104.2, 974	50.5, 478	49.2, 479	55.1, 510	63.6, 583	32.8, 295	225.9, 2124 (451.8, 4248)	200.4, 1889
[NaCl] (mM)	50	50	50	50	50	300	300	50	50	50	500
pH	8.0	8.0	8.5	8.0	8.0	8.0	8.0	7.5	8.0	8.0	8.0
										10% glycérol	2 mM EDTA
											10% glycérol
**DLS**^b^											
*R*_h_ (nm)	7.3 (7.9)	6.9 (8.7)	4.2	4.5	3.4	3.0	3.7	3.4	2.4	6.5 (–)	8.1
Pd (%)	17 (25)	26 (35)	19	23	25	20	15	27	19	13 (–)	18
**SAXS**^c^											
*R*g (nm)	7.4 ± 0.1/7.7 (7.4 ± 0.1/7.6)	8.2 ± 0.4/7.9 (10.3 ± 0.1/10.7± 0.1)	3.8 ± 0.1/4.0	3.8/3.8	2.8 ± 0.1/2.9	2.8 ± 0.0/2.7	3.4 /3.5	3.1/3.4	1.9 ± 0.1/2.0	5.7/5.9 (8.0 ± 0.2/8.4)	6.4 ± 0.3/ 6.5
MW (kDa)	210/214 (248/255)	−/−	105/105	110/110	−/−	−/−	44/44	46/47	26/27	−/−	−/−
		−/−								−/−	
MW_ind_ (kDa)	214 ± 26/190 (216 ± 22/179)	227 ± 30/227 (431 ± 36/290)	116 ± 7/125	102 ± 2/115	54 ± 7/58	54 ± 6/60	58 ± 3/64	59 ± 1/63	27 ± 3/30	221 ± 23/225 (334 ± 99 /343 )	182 ± 16/217
*D*_max_ (nm)	24.8 (24.8)	25.0 (36.0)	12.7	13.0	10.0	8.0	12.0	12.0	5.5	21.0 (29.0)	20.2
Oligom. state	M (M)	M (D)	M	M	M	M	M	M	M	M (D)	M
t*R*_g_ (nm) ^d^	3.8/4.9/13.9	3.8/4.9/13.9	3.1/4.1/ 10.9	3.0/3.9/10.3	2.3/3.0/7.2	2.3/3.0/7.3	2.3/3.0/7.3	2.5/3.2/8.0	1.9/2.5/5.6	4.0/5.3/13.9	3.9/5.0/14.4
*χ* DAMMIN/ GASBOR/											
Comparison with HR model	1.36/2.69/3.11 (−/−/−)	1.16/−/− *(1.16/−/−)*	1.80/2.80/1.02	1.57/1.83/1.36	1.28/1.10/1.11	2.91/2.72/1.14	2.12/2.68/3.10	1.86/4.32/1.97	3.41/2.93/1.67	1.44/1.54/−	1.32/1.56/−

^a^ Theoretical molecular weight and number of residues given for the tagged proteins in the case of Pks13, holo-Pks13, C16-Pks13(S1533A), Mas, PpsA, fACP1‑KS‑AT, fKS‑AT, fKS, fAT, AT52 and after removal of the His_6_-tag for fACP2‑TE and fTE

^b^ Hydrodynamic radii (*R*_h_) and percentage of polydispersity obtained from dynamic light scattering at 20 °C. Histograms of *R*_h_ can be found in Additional file 1: Table S1

^c^*R*_g_, gyration radii based on Guinier plot/distance distribution function. MW, molecular weight based on *I*_*0*_ from Guinier plot/*I*_*0*_ from distance distribution function *p(r)*. MW_ind_, molecular weight based on the concentration-independent methods by Rambo and Tainer/SAXSMoW calculator. *D*_max_ maximum dimension. Oligomeric state (monomer, M; partial dimerization, D) obtained from SAXS (at 12–15 °C). For data arising from online HPLC measurements, only the concentration-independent evaluation of the molecular weight is given. Error values are given except when their mathematical rounding gives 0

^d^ Theoretical radii of gyration calculated for a monomeric globular/dimeric globular/unfolded protein

### Characterization of flexibility and global structural properties

**Fig. 3:**
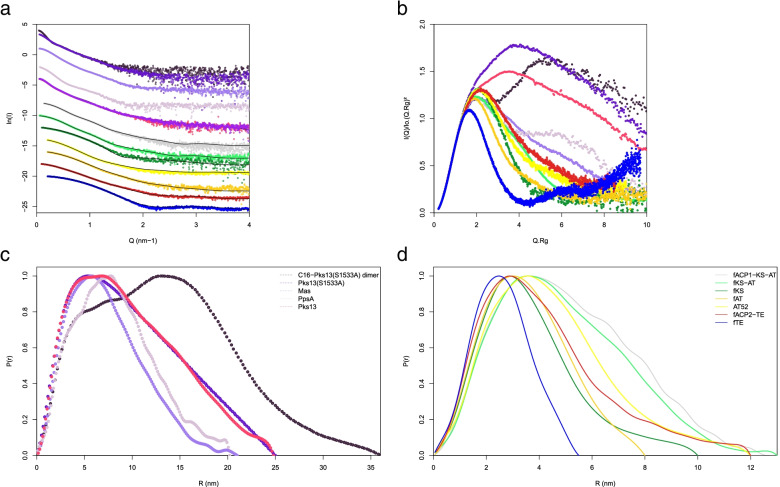
SAXS analysis of Pks13, Mas and PpsA. **a** Experimental scattering curves measured at 50 mM NaCl (except otherwise stated) and for the proteins without the phosphopantetheine arm (except otherwise stated). The logarithm of intensity is displayed as a function of the momentum transfer *Q*. The normalized curve has been displaced downwards by 1 or 2 logarithmic units for clarity, except when precised that they are superposed. The fits obtained from modelling (AllosMod-FoXS or MultiFoXS analysis) are in dark. **b** Normalized Kratky plots, **c**,** d ***p(r)* distance distribution *c*omputed from the experimental scattering patterns. Samples are given in the following order from top to bottom: superposed Pks13(S1533A) monomer (medium-purple) and dimer of C16-Pks13(S1533A) (dark purple), Mas (purple), PpsA (lavender, 500 mM NaCl), superposed Pks13 and holo-Pks13 (i.e. without and with Ppant arms: purple and pink, respectively), fACP1‑KS‑AT (grey), fKS-AT (light green), fKS (dark green), fAT (yellow, 300 mM NaCl), AT52 (light orange, 300 mM NaCl), fACP2-TE (red) and fTE (dark blue)

Preliminary SAXS data collected on full-length Pks13 allowed finding the optimum conditions for monodisperse solutions (Tris-HCl, pH between 7.5 and 8.5, 50 mM NaCl). Following experiments on Pks13 and full-length Mas were then conducted in these conditions. In contrast, 300 mM NaCl was used for conditioning fAT and AT52 to avoid artefactual AT dimerization whereas PpsA was studied at 500 mM to avoid aggregation. The SAXS curves and derived distance distribution functions *p(r)* as well as normalized Kratky plots of all investigated proteins are displayed in Fig. 3. One may observe that the scattering patterns of apo- and holo-Pks13 display negligible differences, indicating that their structures are similar at the resolution probed (Fig. 3a). Normalized Kratky plots were calculated to check for flexibility as described in [52] (Fig. 3b). They show that all Pks13 fragments were folded with relatively low flexibility, a fact that could not be anticipated for example for fACP2-TE, which contains about 135 non-catalytic residues. In contrast, full-length Pks13 displayed Kratky curves corresponding to a flexible entity. This is in line with the characteristic smooth *p(r)* function of Pks13, which highlights the dynamic nature of the full-length enzyme [53]. Pks13(S1355A) displays even more flexibility, though it seems to partly rigidify once loaded by a C16 carbon chain on the catalytic Ser801 of the AT domain, as interdomain correlation peaks appear in the *p(r)* function. SAXS and dynamic light scattering data were used to calculate global structural parameters, e.g. radii of gyration *R*_g_ which were also compared to theoretical values calculated for a monomeric globular/dimeric globular/unfolded protein using equations from [54–56] (Table 1, Additional file 1: Table S2). A rather good internal consistency could be observed. Molecular masses of the proteins in solution were calculated with the usual Guinier procedure and with the analysis of *p(r)* curves. In addition, the concentration-independent methods of Rambo and Tainer and the SAXSMoW calculator were used [57, 58]. Experimental molecular masses are in rather good agreement with values calculated from sequences, the highest discrepancy being for fACP2‑TE and AT52 (Table 1). The latter may be due to inaccuracies in the determination of protein concentration, as a much better agreement was generally obtained with the concentration-independent method.

The molecular mass of full-length Pks13 estimated with the concentration-independent method implemented in SAXSMoW was 190 kDa, which is compatible with a monomer. Mas and PpsA, with estimated molecular mass of 225 and 217 kDa respectively, also mainly form monomers. However, both display a small proportion (less than 10%) of dimers evaluated from the chromatogram at 280 nm (Additional file 1: Fig. S5a, b and d), although the proportion of Mas dimers increased to 24% in 300 mM NaCl (Additional file 1: Fig. S5c). *p(r)* function (Fig. 3c) and *R*_g_ calculation using Guinier analysis show that Pks13 monomer is elongated with a maximum dimension *D*_max_ of 24.8 nm and a *R*_g_ value of 7.7 nm coherent with DLS data, when the theoretical *R*_g_ value for a globular protein of 188 kDa would be about 3.7 nm. PpsA and Mas were also found to be elongated, though less than Pks13, with respective (*D*_max_, *R*_g_) values of (20.2, 6.5) and (21.0, 5.9) nm. PpsA, and to a lesser extent Mas and Pks13, have *p(r)* curves displaying two shoulders. This might indicate that their domains obey to structural segregation. For Pks13 subspecies, the histogram of distances indicates that multiple-domains are more or less elongated whereas the typical bell shape of globular proteins was found for fAT and fTE monodomains.

Pks13(S1533A), which was incubated without C16-CoA to discard an effect of the incubation at 30 °C per se, is monomeric with an overall shape highly similar to that of Pks13, though with a slightly higher *R*_g_ value (7.9 vs. 7.7 nm). We verified that the protein in HEPES buffer was also monomeric. Nonetheless, when the AT catalytic serine is loaded with the C16 chain from the substrate analogue, Pks13(S1533A) undergoes partial dimerization (about 60%, Additional file 1: Fig. S4a). The *R*_g_ and *D*_max_ values increase to respectively 10.7 and 36.0 nm for the dimer (hereafter named C16-Pks13(S1355A) dimer), which eluted in the first peak of the gel filtration.

### Ab initio low-resolution modelling

**Fig. 4:**
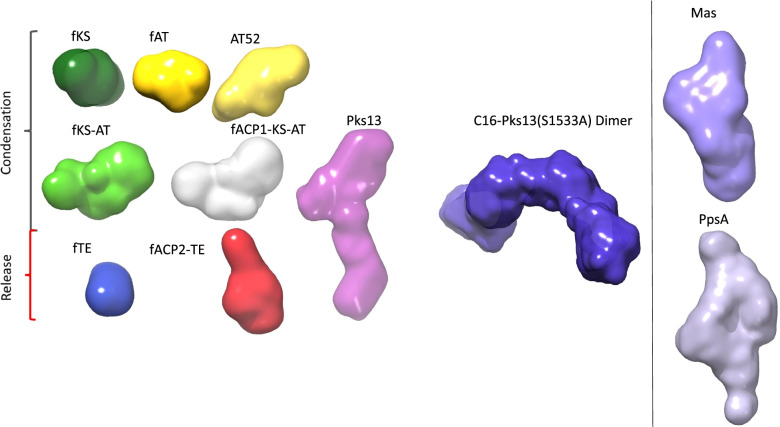
Ab initio models of Pks13 and Pks13 fragments, C16-Pks13(S1533A) dimer, Mas and PpsA. Envelopes were calculated using the GASBOR program except otherwise stated: Pks13 (average envelope, pink) and Pks13 fragments (most probable envelopes: fACP1-KS-AT, grey; fKS-AT, green; fKS, dark green; AT52, yellow; fACP2-TE, red; fTE, blue), C16-Pks13(S1533A) dimer (average DAMMIN envelope, dark purple), Mas (average envelope, purple), PpsA (average envelope, lavender). Images were prepared using the software Chimera (http://www.rbvi.ucsf.edu/chimera)

SAXS data were also used to calculate low-resolution molecular envelopes (Fig. 4 and Additional file 1: Table S2). For each fragment, series of calculations led to similar overall structures, with an average normalized spatial discrepancy below 0.7, vs. 0.9 for entire Pks13 and 1.1 for the C16-Pks13(S1355A) dimer, and *χ* between 1.2 and 3.4 (Table 1). The envelope of the entire Pks13 enzyme is highly elongated and has a two-body shape, with a foot and a head. fKS-AT displays an elongated kinked envelope. fACP1-KS-AT combines in a somehow more globular way whereas fACP2-TE forms a highly elongated structure. Comparing the fACP1-KS-AT and fACP2-TE envelopes with that of the full-length enzyme (Fig. 4, Additional file 1: Fig. S6a) led to a model where the foot and the head of Pks13 are respectively composed of fACP2-TE and fACP1-KS-AT and with the missing 93 residues between AT and ACP2 (i.e. the residues not included in these two fragments) being at the junction. As could be expected from their *D*_max_ and *R*_g_ values, Mas and PpsA are much more compact than Pks13. They also display a two-body shape, in accordance with the fact that both are reducing-PKSs for which segregated condensing and reducing compartments are expected. In line with the doubling of molecular weight, the hydrated volume calculated by DAMMIN for the dimer of C16-Pks13(S1355A) is almost double that of Pks13(S1355A).

### Hybrid modelling combining low- and high-resolution structural information

**Fig. 5:**
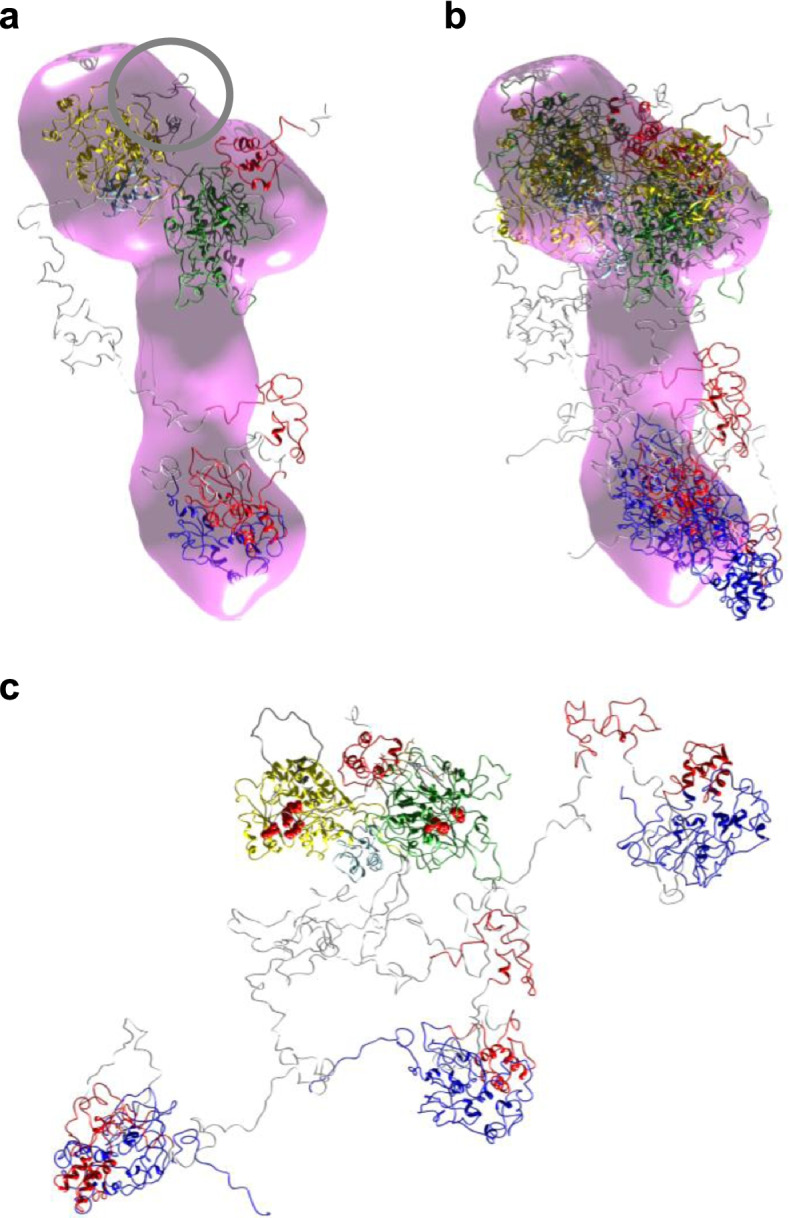
Hybrid modelling of Pks13. **a** With the most probable population superposed on the envelope. The Pks13 insertion is circled in dark grey. **b** With the three populations constituting the minimal ensemble, independently fitted within the envelope. **c** With the three populations aligned via ACP1-KS-AT. Equivalent residues known to interact with the C16 substrate analogue in the complex between AcpP and the ketosynthase FabF (*E. coli*) and residues interacting with CoA of the α-chain in the AT of Pks13 are highlighted in red on the corresponding KS and AT domains. Colour code: ACPs, red; KS, green; AT, yellow; TE, dark blue; linker regions, light grey; Pks13 insertion, dark grey

Hybrid modelling was undertaken for all monomeric Pks13 species. Preliminary comparison of the scattering data for monodomains fKS, fAT, AT52 and fTE to calculated values using the crystal structures of CurL KS domain and those of Pks13 (AT52 and fTE) led to *χ* values of 3.5, 2.1, 24.4 and 2.1, respectively. Rather poor fits were due to missing residues, i.e. residues not resolved in the structures or fragment boundaries that do not fit exactly the corresponding high-resolution structures. Modelling of missing residues with AllosMod-FoXS and MultiFoXS software led to fits of 1.1, 1.1, 6.6/4.1 and 1.7 for fKS, fAT, AT52 (including the N-terminal helix or not) and fTE, respectively (Table 1 and Fig. 3a). With the assumption that the portion of LD included in AT52 could be partly destabilized due to the fragmentation of the highly intertwined KS-AT domain, thus letting this portion mobile relative to the AT domain, the *χ* value decreased down to 3.1. In a similar way, modelling the 80 missing Pks13 residues, corresponding to the 540–620 insertion between KS and LD, using the KS-AT structure from CurL, improved the fit against fKS-AT scattering data from *χ* = 7.7 to 1.4. Two distinct arrangements have been observed so far for the AT domain in PKS structures. They correspond to an ‘AT-down’ conformation, as observed in PikAIII or in DEBS module 1 (though with a distinct molecular mechanism, [27]), and an ‘AT-out’ conformation in other PKSs and FASs [36]. Here, the KS-AT envelope overlaps well with the structure found for the CurL KS-AT didomain, with a normalized spatial discrepancy of 1.4, which may indicate that fKS-AT is close to an ‘AT-out’ conformation. An additional volume on top of LD between KS and AT domains could correspond to the 80-residue-long insertion specific for Pks13 (Fig. 5a). Starting from the AllosMod-FoXS KS-AT model, hybrid modelling of ACP1-KS-AT led to a final *χ* value against fACP1-KS-AT data of 1.0. Superposition of the combined high-resolution information with the SAXS envelope of fACP1-KS-AT revealed that the three catalytic domains are in a compact configuration (Fig. 5a). Hybrid modelling of ACP2-TE led to a *χ* value against fACP2-TE data of 2.0, with two conformation populations, the major one representing 83% of the ensemble, with a *R*_g_ value of 3.4 nm consistent with the low flexibility of this fragment and with the average *R*_g_ value obtained from *p(r)* analysis. fACP2-TE displays an elongated and strikingly straight configuration, where TE is at one extremity of the fragment and ACP2 lies in the middle (Fig. 5a).

As fACP1-KS-AT and fACP2-TE display not at all or low flexibility, entire Pks13 has been modelled considering ACP1-KS-AT and ACP2-TE as rigid bodies (Fig. 5a). The minimal ensemble that best fitted the data led to a *χ* value of 3.1 and is composed of three populations in which the entire ACP2-TE foot of the enzyme is mobile. Two populations predominate with respective *R*_g_ values of 7.1 nm (42% of the ensemble) and 6.2 nm (43%, Fig. 5a). The last population (15 % of the ensemble) has a *R*_g_ value of 8.8 nm. These three populations can be either independently fitted within the envelope of the entire enzyme (Fig. 5b) or after superimposition of their ACP1-KS-AT domain (Fig. 5c), showing the flexibility of the ACP2-TE foot relative to the condensing region. As already mentioned in studies on flexible entities [52], envelope density may then be partially lacking around flexible parts of the high-resolution model, due to averaging over the multiple conformations of this region. In all three populations, the releasing part is rather extended, although a partial flexibility allows ACP2 and TE to go closer to either KS or AT domains (Fig. 5c), two domains ACP2 has to interact with. Other ACPs having a comparable role to ACP2, i.e. shuttling between AT and KS, have been studied in interaction with their cognate catalytic domain. This is the case for the crosslinked complex between AcpP and the ketosynthase FabB and FabF from *E. coli* type II FAS [59], which allows us to position residues known to interact with the C16 chain of the substrate analogue on the KS domain of Pks13. Basic residues that have been shown to interact with CoA of the α-chain [25] also define part of the interface between the AT and ACP2 domains (Fig. 5c).

## Discussion

The first question we wanted to challenge was whether phosphopantetheinylation had an impact on the structure of Pks13. Negligible structural differences could be observed between the apo- and holo-form in the conditions studied here. To the best of our knowledge, this has never been demonstrated for other FASs and PKSs so far. Our strategy also raised the question whether Pks13 fragments represent the native conformation found in the full-length enzyme. In addition to verifying activity whenever possible (i.e. on Pks13 [42], fTE [40] and AT52 [25]), Kratky analysis revealed that most fragments were well folded with weak to intermediate flexibility. Ab initio reconstruction led to the same conclusion. fKS-AT and fACP1-KS-AT displayed little flexibility, which is coherent with the fact that the KS-AT didomain has a complex and intricate scaffold with structured LD in all FAS and PKS structures solved. Nonetheless, in the course of the catalytic cycle, the orientation of the AT domain relative to the KS domain maybe pushed toward a second distinct state [27]. Noticeably, AT52 includes a major part of the long insertion specific of Pks13 found at residues 542–624. In the crystal structure, residues 595–624 form a 16-residue-long α-helix, which wraps around the AT domain [25] whereas residues 576–594 were unresolved in electron density. It seems that these residues do not constitute a flexible unfolded region based on Kratky plots of both fKS-AT and fACP-KS-AT. This is in accordance with the high amount of proline residues (16%) in this region. Nonetheless, Kratky analysis revealed that AT52 displayed some flexibility, which may be due to helix 595–624 swinging in solution which may lead to LD destabilization and may explain the rather poor fit of AT52 SAXS data with the high-resolution structure. In line with this, it is worth mentioning that fAT, which corresponds to the residues resolved in the AT52 structure, displayed a good fit between crystallographic and SAXS data. The ability of this portion of LD to move relative to the AT domain may either be an artefact of fragmentation, or reflect a pivot that may have a functional role during the catalytic cycle in reorienting the AT domain relative to the KS domain, as has been observed in DEBS M1 structure [27]. Concerning fACP2-TE, bioinformatic analysis suggested that this fragment contains two linker regions from 1154 to 1231 (78 residues) and from 1310 to 1444 (135 residues). Taking into account the structure of the cryptic ΨACP (residues 1370–1450), structural information is still lacking for regions 1154–1231 and 1310–1369. Kratky analysis suggested that flexibility of fACP2-TE is low. This is coherent with what has been observed in the structure of VirA module 5, where the structurally uncharacterized 159 residue-long portion of the KS-to-ACP linker displays a rigid structure, leading to an arm-shape architecture for the KS-ACP-ACP fragment [28]. On the contrary, the Men2 ACP2-TE and ACP1-ACP2-TE fragments have turned out to be highly flexible [60]. Nonetheless, these Men2 construct contain only the ACP1-ACP2 and ACP2-TE linkers (respectively 47 and 57 residue long) whereas our fragment fACP2-TE contains a smaller ACP2-ΨACP linker (37 residues, though 23 additional residues are missing as they were unresolved in the structure of ΨACP) and a 78-residue-long portion of the linker between AT and ACP2 (lAT-ACP2_fACP2-TE_). The amount of flexible residues in linkers between ACPs and between ACP and TE are in the range 66–77% as evaluated with MEDUSA tool [61], and about 35% for lAT-ACP2_fACP2-TE_. Flexibility would be needed especially between ACPs and between ACP and TE to fulfil the catalytic cycle in PKSs, though flexibility between ACPs may depend whether these tandem ACPs have functional equivalence or not. On the contrary, a more rigid linker before ACPs could be needed for the ACP and TE domains to be shuttled efficiently at long distances through a quite rigid arm. Finally, our results reveal that most of the linker regions are globally structured and rather rigid. It is possible that, akin other FASs and PKSs, Pks13 linkers may include cryptic catalytic domains, which were not identified by the bioinformatic analysis. In contrast to both fACP1-KS-AT and fACP2-TE, the full-length enzyme displays flexibility, which account for the fact that it did not crystallize despite intensive efforts. As fragments are less flexible than the entire enzyme, it may indicate that the main flexibility pivot occurs at the frontier between the two larger stable fragments ACP1-KS-AT and ACP2-TE. Our results are in line with a structural segregation of the catalytic domains, with one part dedicated to the condensation and the other dedicated to the release of the product. Structural segregation of catalytic domains is consistent with what has been observed for various FAS and PKS structures [1, 9, 11, 13, 30, 31].

Another striking point is that Pks13, Mas and PpsA were found to be (mainly) monomeric (see Additional file 1: Fig. S5a, b, and c) whereas all PKSs and FASs studied until now were shown to form dimers or higher oligomers, mainly via their KS domain, except in particular conditions where mFAS dimers may for instance dissociate [62]. From a functional point of view, it seems that at least dimers are required for enzyme functionality [63]. However, several excised KS domains or KS-AT didomains have been observed in solution as monomers or in equilibrium between monomers and dimers [8, 20, 30, 31, 64, 65]. The dissociation constant for KS may be low [20], and the avidity of other dimerization elements (such as KS N-terminal dimerization helix, docking domain, reducing or thioesterase domains, all having *K*_d_ values in the μM-mM range) maybe needed to push the entire enzymes towards a more stable dimerization. Mas and PpsA do not have any N-terminal helix but contain such reducing domains, and we could have expected to observe them as dimers. Indeed, dimers were observed for the Mas-like PKS [20]. Yet, in our hands, only a small proportion of dimers could be observed and discarded through online HPLC. 10% of Mas dimers was obtained at 50 mM NaCl, and this amount increased to 24% at 300 mM NaCl, despite the fact that the proteins were loaded at 3 mg/ml. This suggests that the *K*_d_ of dimerization is low and that hydrophobic interactions are involved. PpsA being prone to aggregation, we challenged it at low concentration and specific conditions, which could explain the very low amount of dimer. Concerning Pks13, the sole domain that could dimerize seems to be the KS one, without N-terminal dimerization helix. As a matter of fact, the solved TE structure does not suggest dimerization of this domain [43], and Pks13 does not possess the other putative dimerization elements quoted above. It may be that interactions between KS domains are too weak to be detected in the experimental conditions of our study (50 mM NaCl, 12 °C)*.* However, the relative condensase activity of Pks13 at 50 mM NaCl showed an increase of 22% with respect to the activity at 300 mM (Additional file 1: Fig. S7), which means that an effect of the ionic strength to explain the monomeric state can a priori be excluded. Furthermore, KS-AT didomains of PKSs have mainly been observed as dimers, though with an affinity that may be as low as 0.4 mM [20], which classifies their interaction as highly transient. Here, neither fKS nor fKS-AT were found to be dimeric, and this applies to fACP2-TE and fTE as well. On the other hand, fAT and AT52 were studied at 300 mM NaCl to avoid dimerization, which we hypothesized to be due to fragmentation as no dimer of AT domains has been described so far. Thus, one may hypothesize that Pks13, like NRPSs, functions as a monomer (61). A second possibility, though not reported on similar enzymes, is that dimerization may happen following post-translational modifications such as phosphorylation. Third, a more elongated and monomeric conformation of Pks13, compared to Mas and PpsA, could be required to allow proper interactions with the FAS-II complex that synthesizes the final elongation of the meromycolic chain, with the 4’-phosphopantetheinyl transferase PptT (24.7 kDa) and/or with the acyl-AMP ligase FadD32 (69.2 kDa) that loads the meromycolic chain onto Pks13. Such interactions with Pks13 have been shown for the FAS-II complex [66], FadD32 [67], and are mandatory with PptT. Finally, and more probably, the dimerization of Pks13 that is required for condensase activity may be transient and substrate loading may be needed to push towards a more stable dimerization that would allow to enter into the condensation cycle. As a matter of fact, in contrast to ACPs found in type II PKSs, ACPs from type I PKSs are unable to sequester hydrophobic chains in an acyl-binding cavity [68–70]. Then, one may suggest that when the acyl chain is tethered to ACP1, part of it needs to be accommodated near the KS dimerization surface. In addition, KS domains have a substrate-binding channel in the vicinity of the dimer interface [71, 72]. It has also been postulated that a certain amount of flexibility of catalytic domains, either intrinsic or induced, is an important requirement for FASs and PKSs that bind and process long-chain hydrophobic substrates. Such a flexibility has been observed for the *Mtb* FAS-II enoyl-ACP reductase InhA prior to substrate binding [73] and for the *Mtb* condensing enzyme KasA that needs to accommodate C38-C42 acyl chains [74]. Upon substrate binding, the substrate hydrophobic cavity of KasA undergoes an opening. Therefore, for enzymes that load even longer acyl chains, such as Pks13, a monomeric state of the enzyme may be required to assure the possibility for the KS channel to adjust upon binding of unusual 40- to 70-carbon-long aliphatic chains. In this study, we observed that dimerization more strikingly occurs while loading the AT domain with a C16 carbon chain as a substrate analogue. This event leads to increased avidity between Pks13 monomers. Dimerization then occurs, leading to a highly elongated and mostly symmetric Pks13 dimer, even though no symmetry has been imposed in the course of envelope calculation. Though surprising, this allosteric oligomerization may be a piece of a catalytic mechanism that requires loading of the two substrates as the signal for stable dimerization and condensation to proceed. Further studies would be needed to test the effect of the loading of the second substrate, and to model its impact on the enzyme structure.

Hybrid modelling of both fKS-AT and fACP1-KS-AT revealed that despite the 80-residue-long insertion between KS and LD (compared to the corresponding region in other PKSs and FASs), the architecture of Pks13 would be closer to mFAS than PikAIII. The 80-residue-long insertion corresponds to an additional envelope on top of LD within the envelope of fKS-AT (Fig. 5a). This is coherent with a positioning of helix 595–620 as that observed in the structure of AT52 [25]. The insertion might be used as a docking template for FadD32 or to stabilize in some way the long acyl chain of the substrate along its loading on KS. ACP1 would be localized in the vicinity of the KS domain, in an analogous region to the SAT domain in CTB1 [32] and to the docking domain to the upstream ACP in CurL, both SAT and upstream ACP being devoted to the loading of the KS domain. Thus, ACP1 and ACP2 may be located on opposite sides of the KS domain. Such an arrangement has already been observed in DEBS module 3 [31, 75]. In addition, two distinct entrances for the respective delivery of the upstream and downstream ACP-bound substrates have been observed in PikAIII [36], which is also consistent with the ACP1 and ACP2 positions in our Pks13 model. In Pks13, ACP1 is strictly devoted to the loading of KS in contrast to ACP2 that need to interact with AT, KS and TE. This could explain why fACP1-KS-AT displays a compact structure and low flexibility. Our modelling also suggests that ACP2-TE is on the opposite side of KS compared to ACP1. This is coherent with the positioning of ACPs of comparable function in both CT1B and CurL, where the ACP that shuttles the chain being elongated is on the opposite side to the domain that loads the substrate on the KS domain. fACP2-TE appears rigid despite the fact that among the Pks13 subspecies studied here it has the highest amount of linkers, whose structures remain to be solved. It may be that parts of the corresponding residues correspond to cryptic enzymatic domain(s), as observed in other FASs and PKSs. Linker regions of Pks13 may altogether constitute an extensive scaffolding matrix that could be involved in the exact positioning of the catalytic domains and/or enzymatic partners along the catalytic cycle. The pivot for flexibility at the interface between the condensing and termination blocks would allow ACP2 to shuttle between the AT and KS. Modelling of full-length Pks13 allowed us to propose a subset of compatible structures, with the whole ACP2-TE subdomain being able to explore several conformations. This subset is coherent with an average *R*_g_ of 7.7 nm. These structures define swinging motions of ACP2-TE that might allow ACP2 to interact with both KS and AT in the course of the catalytic reaction, though an additional conformational rearrangement of ACP2-TE may be needed once Pks13 is loaded with its substrates. Noticeably, our model of Pks13 is highly distinct from the structure predicted by AlphaFold [76] which proposes a structure much more compact, with an *R*_g_ of 4.5 nm.

## Conclusions

Pks13 in addition to its importance as a target to develop new drugs against tuberculosis is a particularly unusual type I PKS in the sense that (i) it has an atypical domain organization with two ACP domains, (ii) it loads specifically long substrates activated in an unusual way and (iii) it has an atypical product release mode. Our SAXS study highlights that Pks13 is an elongated protein divided into two distinct regions corresponding to the condensation and releasing activities. Apo- and holo-Pks13 have the same overall structure. They both display a quite large flexibility due to a pivot at the interface between the condensing and releasing regions. Our fragmentation study and hybrid modelling, which takes into account enzyme flexibility, allowed us to propose a model in which high-resolution structures of Pks13 domains and structural homologues have been located within the enzyme. In this model, the KS-AT architecture seems close to the more common mFAS architecture. ACP1 is positioned in a region analogous to the position of an upstream ACP in modular PKSs. This analogy between ACP1 and upstream ACPs is also reflected in the restricted motion of ACP1. ACP2 is positioned on the other side of the KS domain, in the strikingly elongated C-terminal region of the enzyme. This C-terminal region might move around the pivot region upstream to ACP2 allowing ACP2 to interact with its AT and KS partner domains. In that sense, ACP2 has a behaviour related to the loosely tethered ACP in mFAS. Strikingly, the enzyme is a monomer in the conditions challenged, which may be required for the interaction with its known partners (e.g. FAS-II complex, FadD32 and PptT) and/or to adjust the long acyl chains it has to condense. Nonetheless, the loading of a 16-carbon-long substrate analogue on the AT domain pushes the enzyme towards dimerization. Overall, this structural information may be critical to discover new drugs with novel mechanisms of action, targeting the interactions between Pks13 and their enzymatic partners or between catalytic domains within Pks13 itself. One possibility, suggested by our study, would be to prevent stable dimerization of the enzyme.

## Methods

### Design of the constructs

#### Bioinformatic analysis

The amino acid sequences of all PKS from *M. tuberculosis* were used to derive combined information for each domain. The various catalytic domains were first located in each PKS using the program SEARCHPKS [77]. Sequences related to the same catalytic domain were then aligned together and with the sequences of structural homologues using the program MultAlin [78]. Domain boundaries were tentatively delineated from the multiple sequence alignments based on identical or conserved catalytic and structural residues and taking into account predicted or observed secondary structure elements. Consensus secondary structures of Pks13 were produced using multiple secondary structure prediction tools: PHD and PROF [79] through the PredictProtein prediction server, the Jpred server [80], the PSIPRED server [81], the Sable 2 server [82], the Porter server [83], SSPro and SSPro8 available at the SCRATCH Protein Predictor [84], nnpredict [85], and SAM-T04 [86]. The program WU-Blast2 as operated by the European Bioinformatics Institute server [87] was used to find regions of sequence similarity from biological structures in the Protein Data Bank. This analysis allowed to propose boundaries for the KS-AT didomain (119–1018), but unfortunately, its production only provided inclusion bodies.

#### Limited proteolysis

Two Pks13 fragments containing respectively the AT (residues 576–1063, which we previously referred to as AT52) and TE (residues 1437–1725, here referred to as fTE) catalytic domains were obtained by limited proteolysis of the full-length protein (Table 1). Experiment details on AT52 and fTE have been previously published [25, 40].

#### Domain trapping

Soluble domains of Pks13 were also identified from random DNA fragments using the split GFP domain trapping method [88]. Briefly, the full-length gene was amplified by conventional PCR using Platinum Taq Polymerase (Invitrogen, Carlsbad, CA) and specific primers. Random digestion of the double-stranded DNA was performed with 0.05 units of deoxyribonuclease I (DNase I) in the presence of 2 mM CoCl_2_. Termini of the cleaved products were polished using 3′–5′exonuclease activity of Vent® polymerase (New England Biolabs, Beverly, MA) and blunt-ligated into the StuI site of a digested DHFR insertion pET vector. The library obtained was selected on 6 μg/ml trimethoprim to eliminate fragments inserted in the wrong open reading frame. The pool of in-frame fragments were subsequently subcloned into the NdeI/BamHI sites of the split GFP vector to screen for protein solubility [89]. Ninety-six fluorescent *E. coli* colonies were picked using the in vivo split GFP screen, and subsequently assayed using the in vitro solubility split GFP screen as described previously [88]. Fragment boundaries were confirmed by DNA sequencing. Selected soluble domains were then subcloned without the GFP 11 tag into the NdeI/BamHI sites of pET expression vectors for evaluating their large-scale purification. Here, we will focus on the Pks13(1154-1720) fragment that contains the C-terminal ACP2-TE didomain of the condensase (referred to as fACP2-TE, Table 1). Other fragments, fACP1-KS (residues 1–630), fAT–ACP2 (residues 576–1383) and fAT-ACP2-TE (residues 576-1733), were challenged but have been found to be unsuitable for this study due to aggregating propensity.

#### Combined approaches

Four other constructs (Table 1) were designed based either on the results obtained in the course of this study (fACP1‑KS‑AT: residues 1–1063) or on the published structures of AT52 [25] (fAT: residues 591-1046) and DEBS module 3 KS-AT [22] (fKS: residues 119–575; fKS-AT: residues 119–1070).

Molecular weights (MW) and extinction coefficients at 280 nm were calculated from amino acid composition using ProtParam [90].

### Expression and purification of *Mtb *Pks13, *M. bovis* BCG PpsA and *M. bovis* BCG Mas full-length proteins, and of *Mtb* Pks13 fragments

The *Mtb* H37Rv *pks13, M. bovis* BCG *ppsA* and *M. bovis* BCG *mas* genes have been amplified by PCR and inserted in a pET26b expression vector (Novagen). These constructions added 13 residues, including a His_6_-tag at the C-terminus, leading to proteins that contain respectively 1746, 1889 and 2124 amino acid residues. The DNAs coding for the various fragments have also been amplified using PCR, then inserted in the pET28a expression vector (Novagen), in order to obtain the fragment genes fused with the sequence coding for a cleavable His_6_-tag at the N-terminus. The corresponding constructs lead to proteins that contain 5–6 extra and 22–23 residues whether the His_6_-tag was cleaved or not. Production and purification of Pks13(S1533A) have already been described in [40].

For expression, plasmids were transformed in *E. coli* BL21(DE3)pLysS strain (fragments fKS-AT, AT52, fAT and fTE), in *E. coli* BL21(DE3)Δ*entD*:pLysS strain, which lacks the *E. coli* EntD PPTase [47] (entire apo-Pks13, Mas and PpsA; fragments fACP1‑KS‑AT, and fACP2‑TE; in co-expression with the phosphopantetheinyl transferase Sfp of *Bacillus subtilis* for holo-Pks13), and in *E. coli* BL21(DE3) for fKS. The transformed strains were grown overnight at 37 °C in 10 ml Terrific Broth (TB) medium containing either kanamycin (30 μg/ ml) or both kanamycin (30 μg/ml) and chloramphenicol (40 μg/ml) when pLysS was present. The precultures were then diluted to an OD_600_ of about 0.01 in the same media containing 30 μg/ml kanamycin and grown at 37 °C (Pks13, fTE and fACP2‑TE), 30 °C (fACP1‑KS‑AT, fKS-AT, AT52 and fAT) or 20 °C (fKS). When the OD_600_ of the cell culture reached 0.6, isopropyl-β-d-thiogalactoside (IPTG) was added at a final concentration of 1 mM (except for fKS, 0.1 mM). After incubation for 4 h (overnight for fKS), cells were washed in 50 mM Tris‑HCl pH 8.0, 150 mM NaCl, harvested by centrifugation (5000×*g* for 15 min), and stored at −20 °C for later use.

Purifications were carried out at 4 °C. The wet cell pastes corresponding to soluble expressed proteins were suspended in lysis buffer (usually 50 mM Tris‑HCl pH 8.0, 300 mM NaCl, 10 mM imidazole, 0.1% Triton X-100, except for Mas and PpsA), sonicated on ice (3 cycles of 30 s sonication and 1 min rest). The cell lysates were centrifuged at 10,000×*g* for 60–90 min at 4 °C. Supernatants were then loaded on a nickel Chelating Fast Flow column (GE Healthcare Life Sciences) pre-equilibrated with buffer A (usually 50 mM Tris‑HCl pH 8.0, 300 mM NaCl, 10 mM imidazole). The column was washed with buffer A until the absorbance at 280 nm was near 0. Then a first step, usually at 20% of buffer B (50 mM Tris‑HCl pH 8.0, 300 mM NaCl, 300 mM imidazole), allowed eluting contaminants. A further step at 100% buffer B, or a gradient between 20 and 100% buffer B, allowed to elute the recombinant proteins of interest. Fractions containing the recombinant proteins were pooled and eventually the His_6_-tag was cleaved using thrombin (Novagen). Then the proteins were concentrated using Vivaspin centrifugal concentrators (Sartorius) and applied to a HiLoad 16/60 Superdex 75 or 200 column (GE Healthcare Life Sciences) pre-equilibrated with 50 mM Tris‑HCl pH 7.5–8.5 containing 50 or 300 mM NaCl except for Mas and PpsA. The gel filtration columns were eluted at 0.5 ml/min. For fACP1‑KS‑AT and entire PpsA, an anion exchange column (UnoQ6, Bio-Rad) was performed respectively before and after the gel filtration and the proteins were eluted respectively at 220 mM and 300 mM within a NaCl linear gradient. Purification buffers were supplemented with 0.2 mM PMSF, 1 mM EDTA and 2 mM DTT when required. Protein purity was checked using 10 or 12% acrylamide SDS-PAGE gels stained with Coomassie Brilliant Blue.

For the analysis of C16-Pks13(S1533A) entities, Pks13(S1533A) protein has been incubated at 3.5 mg/ml 1h30 at 30 °C with a six-fold excess of C16-CoA in 50 mM HEPES pH 7.2, and directly submitted to SEC-SAXS experiment. As a negative control, an analogous incubation without C16-CoA has been undertaken.

### Biochemical and biophysical characterization, SAXS studies

The stability and molecular integrity of the purified proteins were controlled prior to SAXS experiments using SDS-PAGE, analytical gel filtration on Superdex 75/Superdex 200 columns and mass spectrometry analysis (see below). In addition, the dispersity of each protein sample was assessed by dynamic light scattering (DLS) measurements using a DynaPro-MS/X molecular-sizing instrument equipped with a microsampler at 20 °C.

#### Pks13 activity assays

The activity assays were performed in the presence of a radiolabeled fatty acid ([1-^14^C] lauric acid), which was activated into acyl-AMP derivative in the presence of FadD32, and of a synthetic long-chain carboxyacyl-CoA (carboxypalmitoyl-CoA). The carboxypalmitoyl-CoA substrate was synthesized and purified as previously described [42]. FadD32 protein from *Mtb* was produced and purified as described [67]. The mycolic condensation assays were performed in the presence of 40 μM [1-^14^C] lauric acid (55 mCi/mmol) and 40 μM carboxypalmitoyl-CoA in the following medium: 50 mM HEPES, pH 8.0, 8 mM MgCl_2_, 2 mM ATP, 6 μM BSA, 7 mM glucose and 7 mM trehalose. Reactions (total volume: 20 μl) were started by the addition of 1 μM purified FadD32 and 2.6 μM purified phosphopantetheinylated holo-Pks13 protein and incubated for 6 h at 30 °C. The stocks of purified enzymes contained small amounts of NaCl, so the basal NaCl concentration in the reactions was 23 mM. To evaluate the effect of NaCl on Pks13 activity, assays with final NaCl concentrations of 50 and 300 mM were also performed. The reaction media were then treated by alkaline methanolysis and reduction by NaBH_4_, and the condensation products were extracted as previously described [42]. The reaction products were dissolved in diethyl ether and analysed by thin-layer chromatography on Silica Gel G-60 plates eluted with dichloromethane. They were quantified by radiolabeling measurement using a phosphorimager (Variable Mode Imager Typhoon TRIO, Amersham Biosciences) and the ImageQuant version 5.1 software (GE Healthcare).

#### NanoLC-MS/MS

SDS solution was added into 30–50 μg of each protein (unmodified and palmitoylated Pks13) to reach a final concentration of 5%. The protein was reduced with 100 mM Tris (2-carboxyethyl)phosphine (TCEP, Sigma) and alkylated with 2-chloroacetamide (Sigma) at 95 °C for 5 min. Each sample was loaded onto S-trap Micro spin columns (Protifi, USA), according to the manufacturer’s instruction [91] and digested with trypsin (Promega) overnight at 37 °C. Digested peptide extracts were analysed by online nanoLC using an UltiMate 3000 RSLCnano LC system (ThermoScientific) coupled with an Orbitrap Fusion Tribrid mass spectrometer (Thermo Scientific) operating in positive mode. Two microliters of each sample (1 μg, analysed by Pierce quantitative fluoremetric peptide assay) was loaded onto a 300-mm ID 5 mm PepMap C18 pre-column (Thermo Scientific) at 20 ml/min in 2% (v/v) acetonitrile, 0.05% (v/v) trifluoroacetic acid. After 5 min of desalting, peptides were online separated on a 75-mm ID 50 cm C18 column (in-house packed with Reprosil C18-AQ Pur 3 mm resin, Dr. Maisch; Proxeon Biosystems) equilibrated in 90% buffer A (0.2% [v/v] formic acid), with a gradient of 10–30% buffer B (80% [v/v] acetonitrile, 0.2% [v/v] formic acid) for 50 min, then 30–45% for 10 min at 300 nl/min. The instrument was operated in data-dependent acquisition mode using a top-speed approach (cycle time of 3 s). Survey scans MS were acquired in the Orbitrap over 350–1400 m/z with a resolution of 60,000, and a maximum injection time (IT) of 50 ms. The most intense ions (2+ to 6+) were selected at 1.7 m/z with quadrupole and fragmented by Higher Energy Collisional Dissociation (HCD). The monoisotopic precursor selection was turned on, the intensity threshold for fragmentation was set to 25,000 and the normalized collision energy (NCE) was set to 28%. The resulting fragments were analysed in the Orbitrap with a resolution of 15,000. Dynamic exclusion was used within 30 s with a 10 ppm tolerance. The ion at 445.120025 m/z was used as lock mass. The Mascot (Mascot server v2.8.1; http://www.matrixscience.com) database search engine was used for peptide and protein identification. MS/MS spectra were compared to a custom-based database containing the Pks13-His sequence. Mass tolerance for MS and MS/MS was set at 10 ppm and 0.02 Da, respectively. The enzyme selectivity was set to full trypsin with two missed cleavages allowed. Protein modifications were fixed: carbamidomethylation of cysteines, variable oxidation of methionines, variable palmitoylation of serine and variable acetylation of protein N-terminus.

#### Native MS

Prior to native MS analysis, apo-Pks13 was desalted in 200 mM ammonium acetate, pH 7 using Micro Bio-Spin devices (Bio-Rad, Marnes-la-Coquette, France) at a molar concentration of 1 μM. The sample was analysed on a SYNAPT G2-Si mass spectrometer (Waters, Manchester, UK) running in positive ion mode (m/z 1000 to 15,000 Th) and coupled to an automated chip-based nano-electrospray source (Triversa Nanomate, Advion Biosciences, Ithaca, NY, USA). The voltage applied to the chip and the cone voltage were set to 1.6 kV and 150 V, respectively. Sample cone voltage, ion energy and trap collision energy were set to 150 V, −2.5 V and 75 V, respectively. The instrument was calibrated with a 2 mg/ml cesium iodide solution in 50% isopropanol. Raw data were acquired with MassLynx 4.1 (Waters, Manchester, UK) and deconvoluted with UniDec [92] using the following parameters: m/z range: 4000–12,000 Th; subtract curved: 100; Gaussian smoothing: 200; bin every 10 Th; charge range 25–40; mass range 50,000–300,000 Da; sample mass: every 10 Da; use automatic m/z peak width; peak detection range: 500 Da, and peak detection threshold: 0.1.

#### SAXS

Preliminary SAXS experiments were conducted on the X33 camera [93, 94] of the European Molecular Biology Laboratory (EMBL) at the storage ring DORIS III of the Deutsches Elektronen Synchrotron (DESY, Hamburg, Germany). Preliminary data were collected on full-length Pks13 at different pH, ionic strengths and protein concentrations using a linear gas detector [95] with scattering vectors (*Q* = 4πsin(*θ*)/*λ*, where 2*θ* is the scattering angle and *λ* = 0.15 nm the incident X-ray wavelength) ranging from 0.12 to 3.44 nm^−1^. SAXS measurements were then performed using a marresearch mar345 image plate detector, with scattering vectors ranging from about 0.1 to 4.5–5 nm^−1^ for the various fragments. SAXS data were all measured at 12 °C for at least 3 concentrations ranging from about 1 to 18 mg/ml (as derived from measurements of absorbance at 280 nm) of freshly prepared protein solutions with and/or without His_6_-tag. 2 mM of fresh DTT was added to the samples just before data collection to avoid radiation damage. For the linear gas detector, the data collected in 15 successive 1-min frames were analysed for the absence of radiation damage and the successive frames were averaged. For the image plate detector, 2-min exposure data sets were collected to check that no change in the scattering pattern occurred with proceeding time and the data were radially averaged by the program MAR-PRIMUS [96]. Data were normalized and corrected for the detector response, and difference curves after the subtraction of buffer scattering were scaled for protein concentration. All data analysis steps were performed using the program PRIMUS [97, 98]. Some data displayed a slight concentration dependence, in which case composite curves were used, using the lowest concentration in the Guinier zone, and the highest appropriate concentration (Additional file 1: Fig. S8). All Guinier analyses were satisfactory, except for PpsA for which a slight increase at low *Q*-value was observed (Additional file 1: Fig. S9).

Further experiments were performed on the SWING beamline at the SOLEIL synchrotron (*λ* = 1.033 Å). The detector was positioned to collect data with a low-*Q* limit from 0.05 nm^−1^ for the largest entities to 0.20 nm^−1^ for the smallest ones. Both direct (Pks13 and holo-Pks13, Pks13(S1533A) in HEPES, fKS-AT) and eluted samples (Pks13 and holo-Pks13, Pks13(S1533A) incubated with/without C16-CoA, fKS, fAT, Mas, PpsA) from a SEC-3 300 Å Agilent size-exclusion column were conducted into the SAXS flow-through capillary cell at a flow rate of 0.15–0.2 ml/min and a temperature of 12–15 °C. Data were reduced with the in-house FOXTROT application and analysed using PRIMUS.

### Computational aspects

Modelling steps were carried out with programs of the ATSAS and AllosMod-FoXS/MultiFoXS suites [99, 100].

#### Analysis of global biophysical parameters

Forward scattering *I*_*0*_ and the radius of gyration *R*_g_ were first evaluated using the Guinier approximation [101] assuming that at very small angles (*Q* < 1/*R*_g_), intensity may be represented as *I(Q)* = *I*_*0*_ exp[−(*QR*_g_)^2^/3]. Indirect Fourier transform in the program GNOM [102] was used to obtain independent evaluation of both *I*_*0*_ and *R*_g_ from the entire scattering patterns and also provided the distance distribution functions *p(r)* as well as the maximum dimension *D*_max_ of the particles. The molecular mass of the various constructs was calculated by comparing forward scattering of the EMBL data with that for a reference solution of bovine serum albumin (MW = 66 kDa) prepared in 50 mM HEPES pH 7.5. Those of the SWING data were calculated by comparing with a reference solution of lysozyme. Concentration-independent molecular masses were also calculated using both the method of Rambo and Tainer and the SAXSMoW 2.1 calculator using the whole scattering curve [57, 58]. To evaluate an eventual degree of disorder within our constructs, normalized Kratky plots (${\left(Q.{R}_g\right)}^2\frac{I(Q)}{I_0}=f\left(Q.{R}_g\right)$) were calculated [52].

#### Molecular modelling

For each construct, ab initio low-resolution envelopes were generated from experimental X-ray scattering data using the DAMMIN [103] and GASBOR [104] programs using respectively data up to *Q* = 3.0–3.5 nm^‑1^ (about 20 Å resolution; program used in SLOW mode with the default weighting scheme and no particular shape imposed; no symmetry have been imposed for the dimer of Pks13) and 4.48–5.00 nm^‑1^ (about 15 Å resolution). Ten different runs were performed and the corresponding low-resolution models were averaged and filtered at a given cut-off volume using the DAMAVER program suite [105]. In order to position individual domains within multidomain fragments, a multi-phase approach has been used both on fACP1-KS-AT and fACP2-TE with the program MONSA [103] and scattering data up to 2.5 nm^−1^ (25 Å resolution). MONSA is based on the same algorithm as DAMMIN. In addition, it reads multiple data sets and, with the information about the volume fractions of the various phases in the particles, it simultaneously fits the data and provides a low-resolution envelope of each phase within the whole construct. As results were coherent with the following quasi-resolution model (Additional file 1: Fig. S6b), they will not be detailed here.

Attempts to construct a model for multidomain fragments and for the full-length Pks13 were challenged at quasi-high resolution, i.e. using the high-resolution structures of homologues or fragments of Pks13 via BUNCH [106] and MultiFoXS procedures. BUNCH employs simulated annealing to find the optimal positions and orientations of the available high-resolution structures of domains against experimental scattering data, the missing residues (about 370 out of 1733 residues in Pks13) being modelled as chains of dummy residues. On the other side, MultiFoXS first generates a pool of about 10,000 high-resolution structures with the missing residues of the input structure modelled at an atomic level, and then, having the list of residues that are flexible, selects the minimal ensembles that best fit the scattering pattern [99]. It has the advantage compared to BUNCH that non-catalytic linker regions are described at an atomic level rather than as dummy residues and that conformational heterogeneity is addressed explicitly. As results were consistent, only the quasi-high-resolution output from the MultiFoXS server has been detailed here.

#### Comparison with structural homologues

The scattering curves from the high-resolution atomic models of known structures or structural homologues were calculated with the program CRYSOL [107] and compared to experimental data sets using the whole scattering range. Comparative analysis of the high-resolution atomic structures and the low-resolution models obtained from solution scattering was carried out using the program SUPCOMB [108]. The high-resolution structural homologues used were as follows: the ACP1 domain of Pks13 whose structure is accessible via the protein data bank (PDB code 6D8I and 6D8J, residues 10–93; 6C4Q residues 11–95); the KS domain from module CurL (4MZ0 [109], residues 41–463; 43.5% identity and 62.6% similarity with fKS); the AT domain of Pks13, whose structure contains part of KS to AT and post AT linkers (3TZX [25], residues 595–1059), the KS‑AT didomain of module CurL from the curacin A polyketide synthase (4MZ0, residues 41–936, 33.8% identity and 52.1% similarity with fKS-AT); the acyl carrier protein from *E. coli* (1ACP [110], residues 1–77; 28% identity and 48% similarity with ACP2); the thioesterase domain of Pks13 (5V3W [43], residues 1448–1733). In addition, KS of CurL has the highest sequence identity with KS of Pks13, and was used to build a model of KS of Pks13 using I-TASSER [111]. It should be noted that the structure of one part of the ACP2-to-TE linker of Pks13 has been solved and has the fold of an ACP, though lacking the catalytic serine (cryptic ψACP, 6C4V, residues 1370–1450). This structure has also been exploited.

## Supplementary Information

**Additional file 1: Figure S1**. Mycolic acid condensation by Pks13. **Figure S2**. Native mass spectrometry analysis of Pks13. **Figure S3**. Bottom-up proteomic analysis of Pks13(S1533A) incubated without C16-CoA. **Figure S4**. Bottom-up proteomic analysis of Pks13(S1533A) incubated with C16-CoA. **Figure S5**. Gel filtration chromatograms of full-length enzymes. **Figure S6**. Comparison of the Pks13, fACP1-KS-AT and fACP2-TE envelopes. **Figure S7**. Impact of ionic strength on Pks13 activity. **Figure S8**. Analysis of concentration dependence for SAXS data collected in batch mode. **Figure S9**. Guinier analysis and plot of ln I(S) versus ln(S) for the various constructs. **Table S1**. DLS analysis of the various Pks13 entities studied. **Table S2**. Essential details about samples, SAXS data acquisition and analysis, modelling fitting and software used.

## Data Availability

All data generated or analysed during this study are included in this published article, its supplementary information files and publicly available repositories. The SAXS data have been deposited on SASBDB database [112, 113] with the identifiers SASDNK9, SASDNL9, SASDNM9, SASDNN9, SASDNP9, SASDNQ9, SASDNR9, SASDNS9, SASDNT9, SASDNU9, SASDNV9 (https://www.sasbdb.org). The mass spectrometry proteomics data have been deposited to the ProteomeXchange Consortium via the PRIDE [114] partner repository with the dataset identifier PXD029754 (https://www.ebi.ac.uk/pride/).

## Abbreviations

ACP: Acyl carrier protein
AT: Acyltransferase
BCG: Bacillus Calmette-Guerin
CoA: Coenzyme A
DH: Dehydratase
DLS: Dynamic light scattering
ER: Enoylreductase
f: Fragment
FAS: Fatty acid synthase
KR: Ketoreductase
KS: Ketosynthase
LD: Linker domain
m: Mammalian
Mas: Mycocerosic acid synthase
MPT: Malonyl/palmitoyl transferase
MT: Methyltransferase
*Mtb*: *Mycobacterium tuberculosis*
PKS: Polyketide synthase
Ppant: Phophopantetheine
PpsA: Phenolphthiocerol synthase A
SAXS: Small-angle X-ray scattering
TE: Thioesterase
