# Supplementary material for: Solution structure of the type I polyketide synthase Pks13 from Mycobacterium tuberculosis
Source: BMC Biol. 2022 Jun 21;20:147. doi: 10.1186/s12915-022-01337-9 (PMC9210659; doi:10.1186/s12915-022-01337-9)
Supplement: Supplementary file 1 — Additional file 1: Figure S1. Mycolic acid condensation by Pks13. Figure S2. Native mass spectrometry analysis of Pks13. Figure S3. Bottom-up proteomic analysis of Pks13(S1533A) incubated without C16-CoA. Figure S4. Bottom-up proteomic analysis of Pks13(S1533A) incubated with C16-CoA. Figure S5. Gel filtration chromatograms of full-length enzymes. Figure S6. Comparison of the Pks13, fACP1-KS-AT and fACP2-TE envelopes. Figure S7. Impact of ionic strength on Pks13 activity. Figure S8. Analysis of concentration dependence for SAXS data collected in batch mode. Figure S9. Guinier analysis and plot of ln I(S) versus ln(S) for the various constructs. Table S1. DLS analysis of the various Pks13 entities studied. Table S2. Essential details about samples, SAXS data acquisition and analysis, modelling fitting and software used. [file 12915_2022_1337_MOESM1_ESM.docx]

**Supplementary information**

**Solution structure of the type I polyketide synthase Pks13 from *Mycobacterium tuberculosis***

Cécile Bon^1*^, Stéphanie Cabantous^1,2,3^, Sylviane Julien^1^, Valérie Guillet^1^, Christian Chalut^1^, Julie Rima^1^, Yoann Brison^1,4^, Wladimir Malaga^1^, Angelique Sanchez-Dafun^1^, Sabine Gavalda^1,5^, Annaïk Quémard^1^, Julien Marcoux^1^, Geoffrey S. Waldo^2^, Christophe Guilhot^1^ and Lionel Mourey^1*^

Correspondence: cecile.bon@ipbs.fr, lionel.mourey@ipbs.fr

^1^Institut de Pharmacologie et de Biologie Structurale, IPBS, Université de Toulouse, CNRS, UPS, Toulouse, France. ^2^Los Alamos National Laboratory, Bioscience Division B-N2, Los Alamos, NM 87545, USA. ^3^Present address: Centre de Recherche en Cancérologie de Toulouse (CRCT), Inserm, Université de Toulouse, CNRS, UPS, Toulouse, France. ^4^Present address: Toulouse White Biotechnology, 31400 Toulouse, France. ^5^Present address: Carbios, Biopole Clermont Limagne, 63360 Saint-Beauzire, France.

**Table S1.** DLS analysis of the various Pks13 entities studied (part 1/2).

| Pks13 entity | *R*_h_ (nm) | Pd (%) | Mass (%) | Autocorrelation function | Size distribution |
| --- | --- | --- | --- | --- | --- |
| Apo-Pks13 | 7.3 | 17 | 96 |  |  |
| Holo-Pks13 | 7.9 | 25 | 99 |  |  |
| ApoP-ks13(S1533A)  with C16-CoA  (dimer peak) | 8.7 | 35 | 99 |  |  |
| Apo-Pks13(S1533A)  (monomer) | 6.9 | 26 | 100 |  |  |
| fACP1‑KS‑AT | 4.2 | 19 | 100 |  |  |
| fKS‑AT | 4.5 | 23 | 98 |  |  |
| fKS | 3.4 | 25 | 62 |  |  |
| fAT | 3.0 | 20 | 99 |  |  |

**Table S1.** DLS analysis of the various Pks13 entities studied (part 2/2).

| Pks13 entity | *R*_h_(nm) | Pd(%) | Mass (%) | Autocorrelation function | Size distribution |
| --- | --- | --- | --- | --- | --- |
| AT52  (300 mM NaCl) | 3.7 | 15 | 100 |  |  |
| fACP2-TE | 3.4 | 27 | 100 |  |  |
| fTE | 2.4 | 19 | 100 |  |  |
| Mas | 6.5 | 13 | 97 |  |  |
| PpsA | 8.1 | 18 | 77 |  |  |

**Table S2.** Essential details about samples, SAXS data acquisition and analysis, modelling fitting and software used (part 1/3).

| **(a) Sample details** | Pks13  (Holo-Pks13) | Pks13(S1533A)  monomer  (C16-Pks13(S1533A) dimer) | Pks13(S1533A) in HEPES buffer | | fACP1‑KS‑ACP | fKS‑AT | fKS | fAT | AT52 | fACP2‑TE | fTE | Mas | PpsA |
| --- | --- | --- | --- | --- | --- | --- | --- | --- | --- | --- | --- | --- | --- |
| Organism | *Mtb H37Rv* | | | | | | | | | | | *M. bovis BCG* | |
| Source | *E. coli* expressed | | | | | | | | | | | | |
| UniProt sequence ID Construct boundaries  His_6_ Tag  MW (kDa) | I6X8D2  1-1733  Cter  188.0 (188.6) | | | | I6X8D2  1-1063  Tag Nter  115.8 | I6X8D2  119-1071  Tag Nter  104.2 | I6X8D2  119-575  Tag Nter  50.5 | I6X8D2  591-1046  Tag Nter  51.2 | I6X8D2  576-1063  Tag Nter  55.1 | I6X8D2  1154-1733  Untagged  63.6 | I6X8D2 1437-1725  Untagged  32.8 | Q02251  1-2111  Untagged  225.9 | A0A0H3M7U0  1-1876  Untagged  200.4 |
| A_280_, 0.1 % (w/v)  $\bar{\nu}$ (cm^3^ g^-1^)  ρ*_M_* (10^10^ cm^-2^)  ρ*_S_* (10^10^ cm^-2^)  $\Delta\overline{\rho}$(10^10^ cm^-2^) | 0.929 (id)  0.736 (id)  12.307 (12.311)  9.431 (id)  2.876 (2.879) |  | |  | 0.846  0.736  12.304  9.431  2.872 | 0.835  0.737  12.292  9.431  2.860 | 0.789  0.735  12.320  9.431  2.889 | 0.811  0.740  12.257  9.509  2.748 | 0.742  0.739  12.265  9.509  2.756 | 1.008  0.736  12.312  9.431,  2.881 | 1.125  0.739  12.267, 9.431  2.836 | 0.994  0.737  12.298  11.905  0.393 | 1.009  9.737  12.289  12.044  0.245 |
| **SEC-SAXS**  Column  C_loading_ (mg/ml)  V_injection_ (µl)  Flow rate (ml/min) |  | SEC-3 300 Å  3.5  40  0.150 | |  |  |  | SEC-3 300 Å  3  40  0.2 | SEC-3 300 Å  3  40  0.2 |  |  |  | SEC-3 300 Å  3  40  0.15 | SEC-3 300 Å  1.5  60  0.2 |
| **Batch-SAXS**  Concentration range (mg/ml) | 2.0-10.4 |  | | 0.9-2.1 | 3.2-8.0 | 1.2-11.0 |  |  | 0.7-9.6 | 1.0-12.6 | 1.0-10.3 |  |  |
| Solvent source | Last-step dialysis | SEC flow-through prior to elution of protein | | Last-step dialysis | Last-step dialysis | Last-step  dialysis | SEC flow-through prior to elution of protein | SEC flow-through prior to elution of protein | Last-step dialysis | Last-step dialysis | Last-step dialysis | SEC flow-through prior to elution of protein | SEC flow-through prior to elution of protein |

MW, molecular weight from amino acid composition; A280, absorbance at 280 nm; $\bar{\nu}$, partial specific volume from amino acid composition; $\rho_{M}$and $\rho_{S}$ : mean solute and solvent scattering length densities, respectively; $\Delta\overline{\rho}$ mean scattering contrast, estimated using MULCh v1.1.1 https://smb-research.smb.usyd.edu.au/NCVWeb/input.jsp.

**Table S2.** Essential details about samples, SAXS data acquisition and analysis, modelling fitting and software used (part 2/3).

| **(b) SAXS data collection parameters** | Pks13  (Holo-Pks13) | Pks13(S1533A)  incubated w/wo C16-CoA | Pks13 in HEPES buffer | fACP1‑KS‑AT | fKS‑AT | fKS | fAT | AT52 | fACP2‑TE | fTE | Mas | PpsA |
| --- | --- | --- | --- | --- | --- | --- | --- | --- | --- | --- | --- | --- |
| Source  Detector | SWING beamline at SOLEIL  CCD-based AVIEX | | | X33 beamline at DESY-EMBL  MAR345 | SWING beamline at SOLEIL  CCD-based AVIEX | | | X33 beamline at DESY-EMBL  MAR345 | | | SWING beamline at SOLEIL  CCD-based AVIEX | |
| Beam geometry (mm^2^)  Wavelength (Å) | 0.8 × 0.15  1.033 | | | 2 × 0.6  1.5 | 0.8 × 0.15  1.033 | | | 2 × 0.6  1.5 | | | 0.8 × 0.15  1.033 | |
| *Q*-range (nm^-1^) | 0.048-5.000 | 0.049-4.360 | 0.056-4.344 | 0.133-4.984 | 0.069-5.000 | 0.091-5.842 | 0.199-4.350 | 0.199-4.35 | 0.009-4.846 | 0.101-4.852 | 0.069-4.356 | 0.056-6.148 |
| Temperature (K) | 285 | 285 | 285 | 285 | 285 | 288 | 285 | 285 | 285 | 285 | 285 | 285 |
| Exposure time (s) Number of frames  Method for monitoring radiation damage | 0.5  50  frame-by-frame comparison | Continuous 1.5 s data measurements of SEC elution  frame-by-frame comparison | 0.5  50  frame-by-frame comparison | 2 mn  15 | Continuous 1.5 s data measurements of SEC elution  frame-by-frame comparison | | | 2 mn  15 | 2 mn  15 | 2 mn  15 | Continuous 1.5 s data measurements of SEC elution  data frame-by-frame comparison | |
| Absolute scaling method  Normalization | Comparison with scattering from 1 mm pure H2O  To transmitted intensity by beam-stop counter | | | To transmitted intensity by beam-stop counter | Comparison with scattering from 1 mm pure H2O  To transmitted intensity by beam-stop counter | | | To transmitted intensity by beam-stop counter | | | Comparison with scattering from 1 mm pure H2O  To transmitted intensity by beam-stop counter | |

**Table S2.** Essential details about samples, SAXS data acquisition and analysis, modelling fitting and software used (part 3/3).

| **(c) Data reduction, analysis and interpretation** | Pks13  (Holo-Pks13) | Pks13(S1533A) monomer  (C16-Pks13(S1533A)  dimer) | Pks13(S1533A)  in HEPES buffer | fACP1‑KS‑AT | fKS‑AT | fKS | fAT | AT52 | fACP2‑TE | fTE | Mas monomer  (dimer) | PpsA |
| --- | --- | --- | --- | --- | --- | --- | --- | --- | --- | --- | --- | --- |
| Data reduction  Software  Mean relative errors until 3 nm^-1^ (%) | FOXTROT  5.6 (3.7) | FOXTROT  7.7 (9.2) | FOXTROT  9.6 | PRIMUS  6.3 | FOXTROT  9.3 | FOXTROT  29 | FOXTROT  10.3 | PRIMUS  4.2 | PRIMUS  6.5 | PRIMUS  3.2 | FOXTROT  9.8 | FOXTROT  20.1 |
| Basic analyses  Guinier Fidelity^1^ (%)  *R*_g_ (nm)  Io  *p(r)* total estimate  *R*_g_ (nm)    Io | 98 (100)  7.4 ± 0.1  (7.4 ± 0.1)  0.430 ± 0.002  (0.434 ± 0.001)  0.621 (0.623)  7.7 ± 0.1  (7.6 ± 0.1)  0.434 ± 0.001  (0.434 ± 0.001) | 81 (97)  8.2 ± 0.4  (10.3 ± 0.1)  0.094 ± 0.001  (0.075 ± 0.001)  0.569 (0.541)  7.9 ± 0.0  (10.7 ± 0.1)  0.090± 0.000  (0.076± 0.000) | 98  8.5 ± 0.2  0.430 ± 0.001  0.542  8.4 ± 0.0  0.419 ± 0.002 | 100  3.8 ± 0.1  306 ± 0  0.666  4.0 ± 0.0  311 ± 0 | 100  3.8 ± 0.0  230 ± 0  0.623  3.8 ± 0.0  235 ± 0 | 77  2.8 ± 0.1  0.022± 0.000  0.641  2.9 ± 0.0  0.022 ± 0.000 | 96  2.8 ± 0.0  0.110± 0.000  0.660  2.7 ± 0.0  0.108 ± 0.000 | 85  3.3 ± 0.0  0.140 ± 0.000  0.694  3.5 ± 0.0  0.138 ± 0.001 | 79  3.1 ± 0.0  (932 ± 2) 10^4^  0.554  3.4 ± 0.0  (964 ± 1) 10^4^ | 91  1.9 ± 0.1  (739± 1).10^3^  0.665  2.0 ± 0.0  (748 ± 0).10^3^ | 94 (67)  5.7 ± 0.0  (8.2 ± 0.2)  0.130 ± 0.000  (0.086 ± 0.000)  0.658 (0.619)  5.9 ± 0.0  (8.4 ± 0.2)  0.128 ± 0  (0.086± 0.000) | 78  6.5 ± 0.1  0.036 ± 0.000  0.767  6.5 ± 0.0  0.036 ± 0.000 |
| **Shape modelling**  **DAMMIN**  χ  Total excluded DAM volume (10^3^ nm^3^)  CorMap p-value (nm^-1^)  **GASBOR**  χ  Total excluded DRM volume (10^3^ nm^3^) | 1.2 (2.7)  524 (491)  0.2067 (0.6147)  3.0  1.9  297 | 1.2 (1.2)  630 (1000)  0,0347(0,1399)  2.5  -  - | -  -  -  -  -  - | 1.8  210  0.0042  3.5  2.4  184 | 1.6  113  0.1258  3.5  1.8  166 | 1.3  89  0.0787  3  1.1  81 | 2.9  88  0.0000  3.5  2.7  81 | 2.1  154  0.0019  3.5  3.1  84 | 1.9  99  0.035  3.5  4.3  99 | 3.4  45  0.0000  3.5  2.9  50 | 1.4  372  0.0071  3  1.5  361 | 1.8  470  0.5687  3.5  1.7  368 |
| **Missing sequence modelling**  AllosMod-FoXS^2^ χ  MultiFoXS^3^ χ (9)  c1, c2  Population: Rg (nm), % | MultiFoXS  3.11  1.05, 1.93  1: 7.1, 42 %  2: 6.2, 43 %  3: 8.8, 14 % | - | - | AllosMod-FoXS  1.02 | AllosMod-FoXS  1.36 | AllosMod-FoXS  1.15 | AllosMod-FoXS  1.14 | MultiFoXS  3.14  1.05, 4.0  1: 2.7, 42 %  2: 3.3, 15 %  3: 2.6, 17 %  4: 3.5, 26 % | MultiFoXS  1.97  1.0, -0.5  1: 3.4, 83 %  2: 6.7, 17 % | AllosMod-FoXS  1.67 | - | - |

^1^: *Q*.*R*_g_ < 1 for elongated proteins (Pks13, C16-Pks13(S1355A), Pks13(S1355A), fKS-AT, fACP2-TE) and *Q*.*R*_g_ <1.3 for all other species. Figures of the Guinier analysis can be found in Additional File 1: Fig. S9. ^2^AllosMod-FoXS via web server (https://modbase.compbio.ucsf.edu/allosmod-foxs/).  ^3^MultiFoXS via web server (https://modbase.compbio.ucsf.edu/multifoxs/).

**
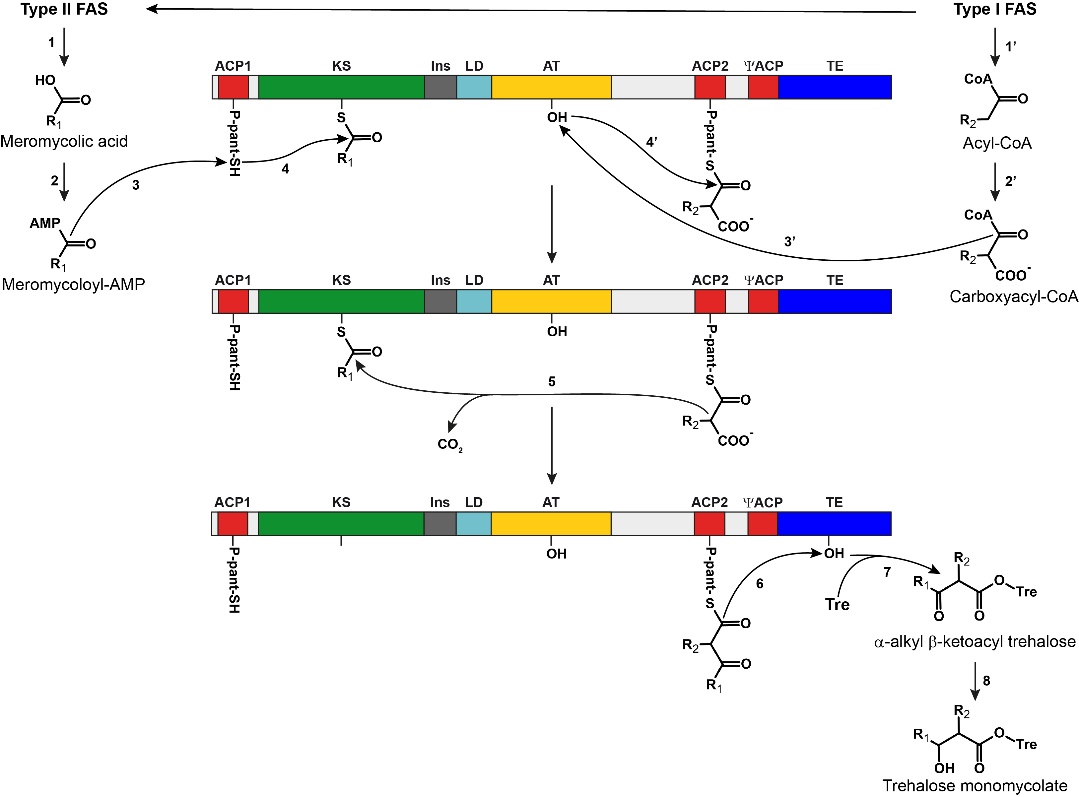
**

**Fig. S1.** Mycolic acid condensation by Pks13. Acyl-CoA and meromycolic acid precursors are produced by type I and type II FAS systems (step 1 and 1’, respectively). Meromycolic acids are activated into meromycoloyl-AMP by the fatty acyl-AMP ligase FadD32 (step 2) which also assists loading onto the N-terminal ACP domain (step 3). The acyl chain is further transferred onto the KS domain (step 4). Acyl-CoA produced by type I FAS are carboxylated by a long-chain acyl-CoA carboxylase complex (step 2’) and the resulting carboxyacyl chain is selected by the AT domain (step 3’) which ensures its transfer onto the C-terminal ACP domain (step 4’). Condensation through Claisen reaction occurs within the KS domain (step 5) and the resulting α-alkyl β-ketoacyl product bound to the C-terminal ACP is loaded onto the TE domain (step 6). After transfer onto a trehalose molecule (step 7) leading to an α-alkyl β-ketoacyl trehalose, the β-keto function of the modified trehalose intermediate is reduced by the CmrA enzyme to form the final trehalose monomycolate (step 8).


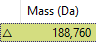

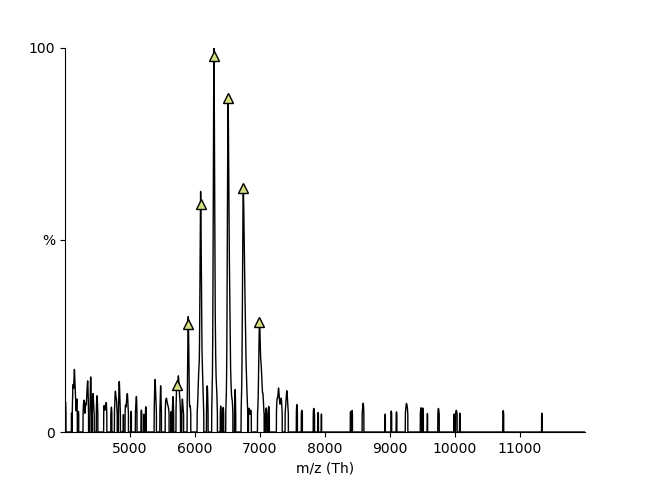

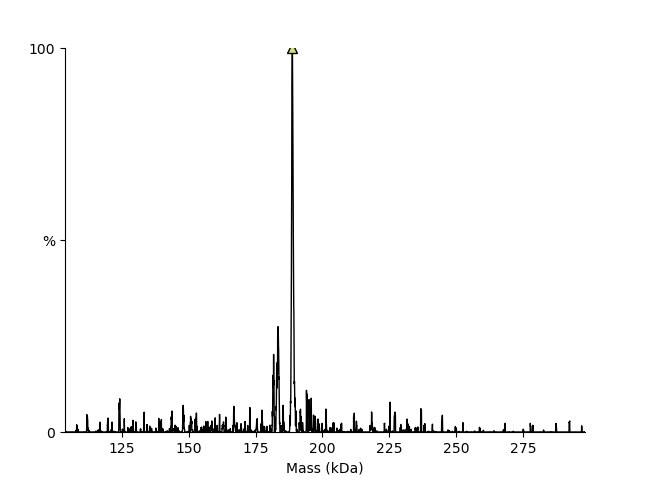


**Fig. S2.** Native mass spectrometry analysis of Pks13. Native mass spectrum of Pks13 before (left) and after (right) deconvolution, showing a single species at 188.8 kDa, in good agreement with the MW of monomeric Pks13 (188.0 kDa).


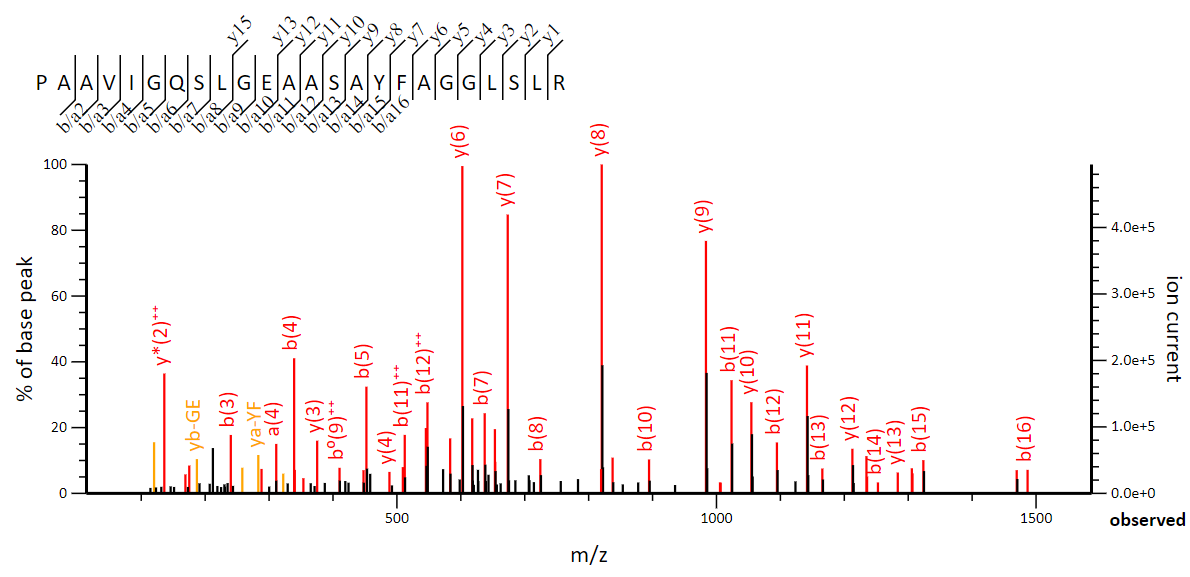

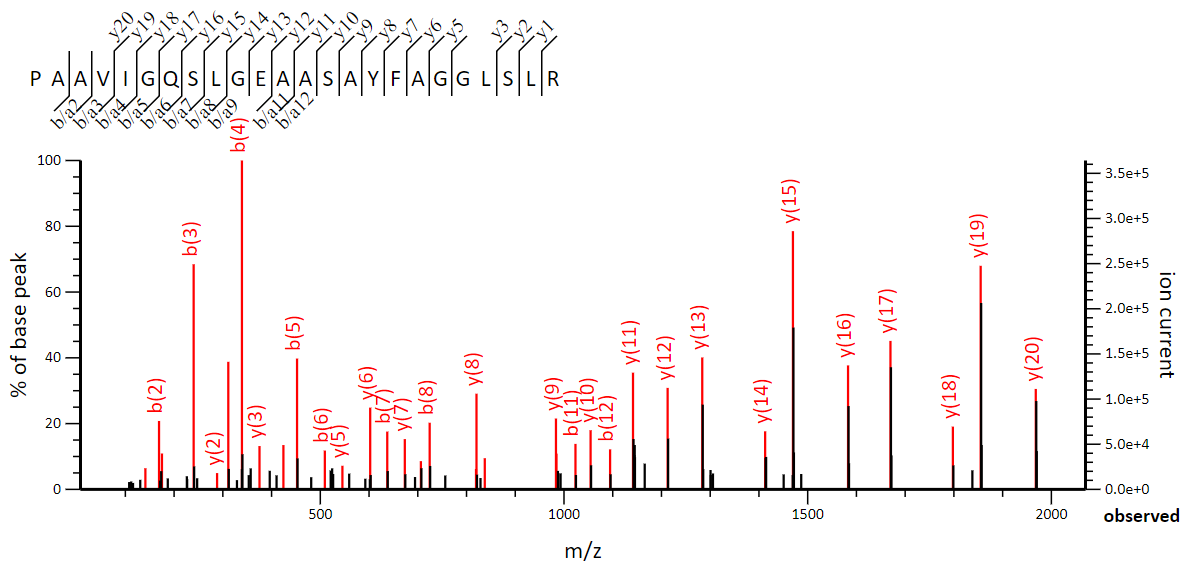

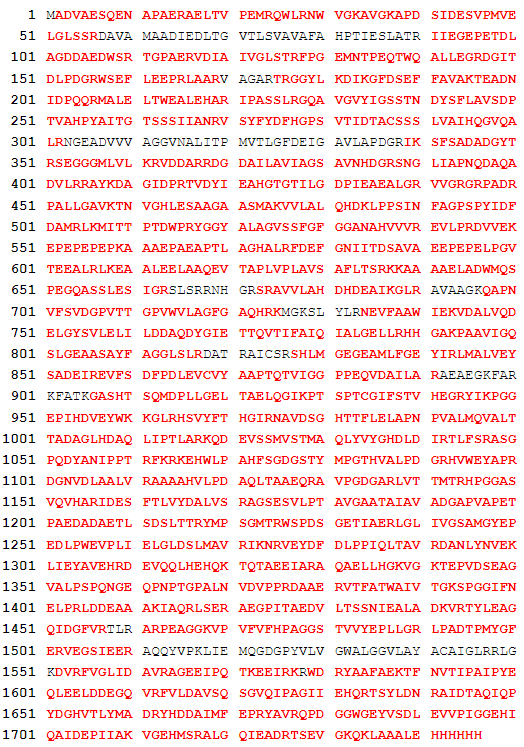

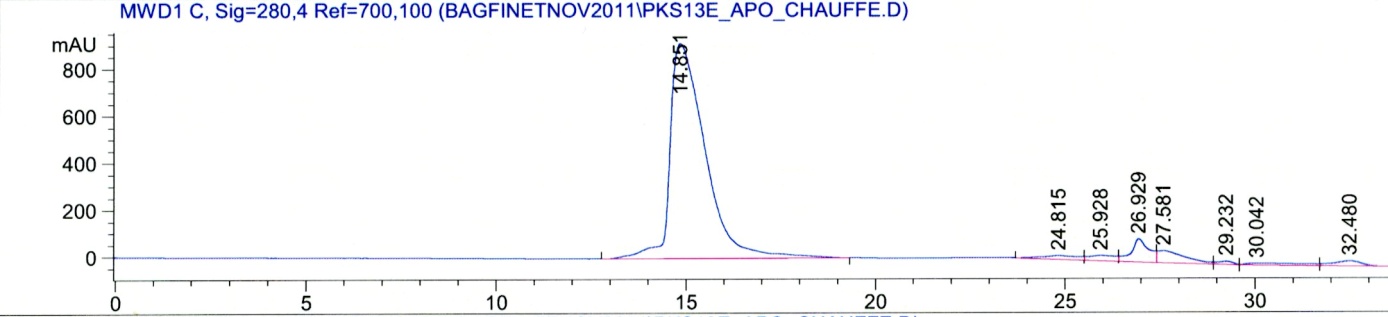


mn

**a**

**b**

**c**

**d**

**Fig. S3.** Bottom-up proteomic analysis of Pks13(S1533A) incubated without C16-CoA. (a) Gel filtration chromatogram (SEC-3 300 Å Agilent, 50 mM NaCl, Tris-HCl pH 8.0, 2 mM DTT, 12 °C). (b) Sequence coverage obtained (90%) and representative MS-MS spectra of (c) doubly (m/z 1153.6111) and (d) triply (m/z 769.4098) charged peptide [794-817] encompassing the unmodified Ser801.

min


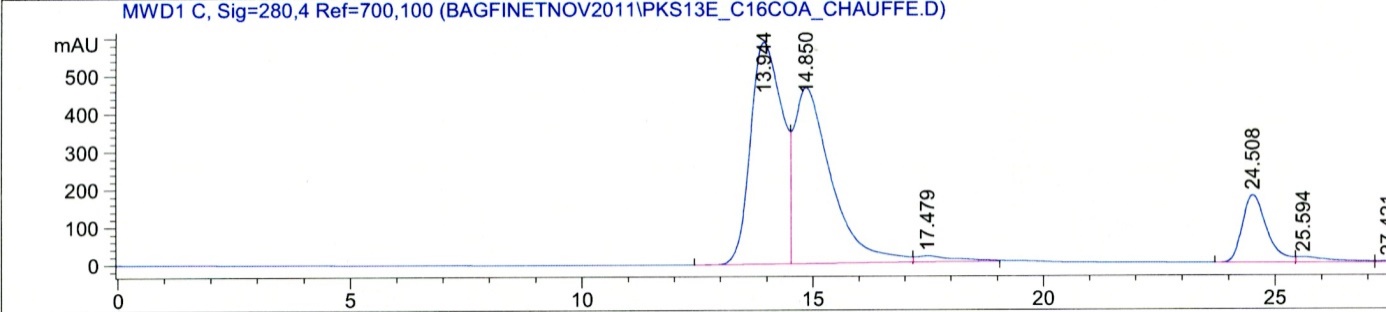

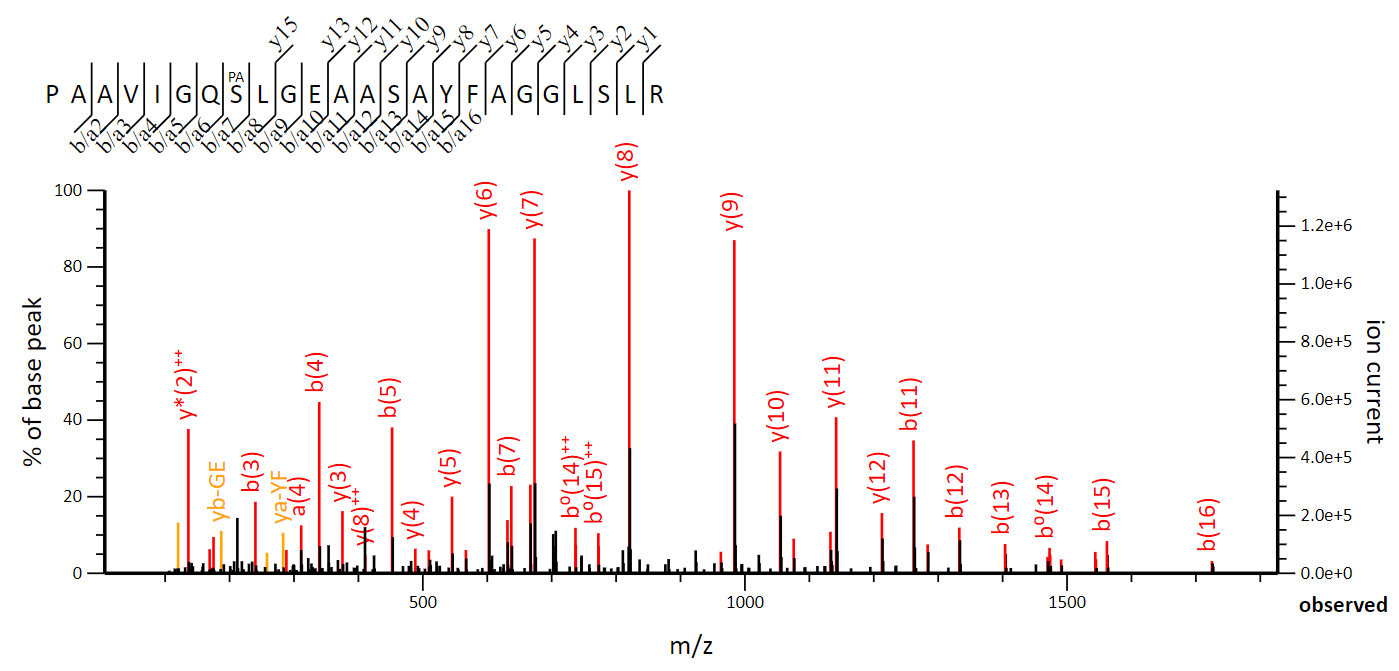


**a**

**b**

**Fig. S4.** Bottom-up proteomic analysis of Pks13(S1533A) incubated with C16-CoA. (a) Gel filtration chromatogram (SEC-3 300 Å Agilent, 50 mM NaCl, Tris-HCl pH 8.0, 2 mM DTT, 12 °C). From this chromatogram a proportion of 60% of dimer has been evaluated. (b) Representative MS-MS spectrum of triply charged (m/z 848.8196) peptide [794-817] encompassing the palmiloylated Ser801.


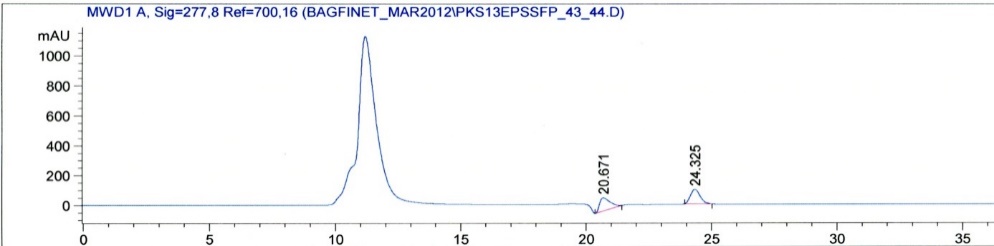


**min**

**d**

**min**

**c**

**min**

**min**

**b**

**a**

**Fig. S5.** Gel filtration chromatograms of full-length enzymes. (a) Pks13 in 50 mM NaCl, Tris-HCl pH 8.0, 2 mM DTT (absorbance at 280 nm, SEC-3 300 Å Agilent, 0.200 ml/mn, 12 °C). (b) BCG Mas in 50 mM NaCl, Tris-HCl pH 8.0, 10% glycerol, 2 mM DTT (absorbance at 280 nm, SEC-3 300 Å Agilent, 0.150 ml/mn, 12 °C). From this chromatogram a proportion of 10% of dimer has been evaluated. (c) BCG Mas in 300 mM NaCl, Tris-HCl pH 8.0, 10% glycerol, 2 mM DTT (absorbance at 280 nm, SEC-3 300 Å Agilent, 0.150 ml/mn, 12 °C). From this chromatogram a proportion of 24% of dimer has been evaluated. (d) BCG PpsA in 500 mM NaCl, Tris-HCl pH 8.0, 10% glycerol, 2 mM EDTA, 2 mM DTT. (absorbance at 280 nm, SEC-3 300 Å Agilent, 0.200 ml/mn, 12 °C).


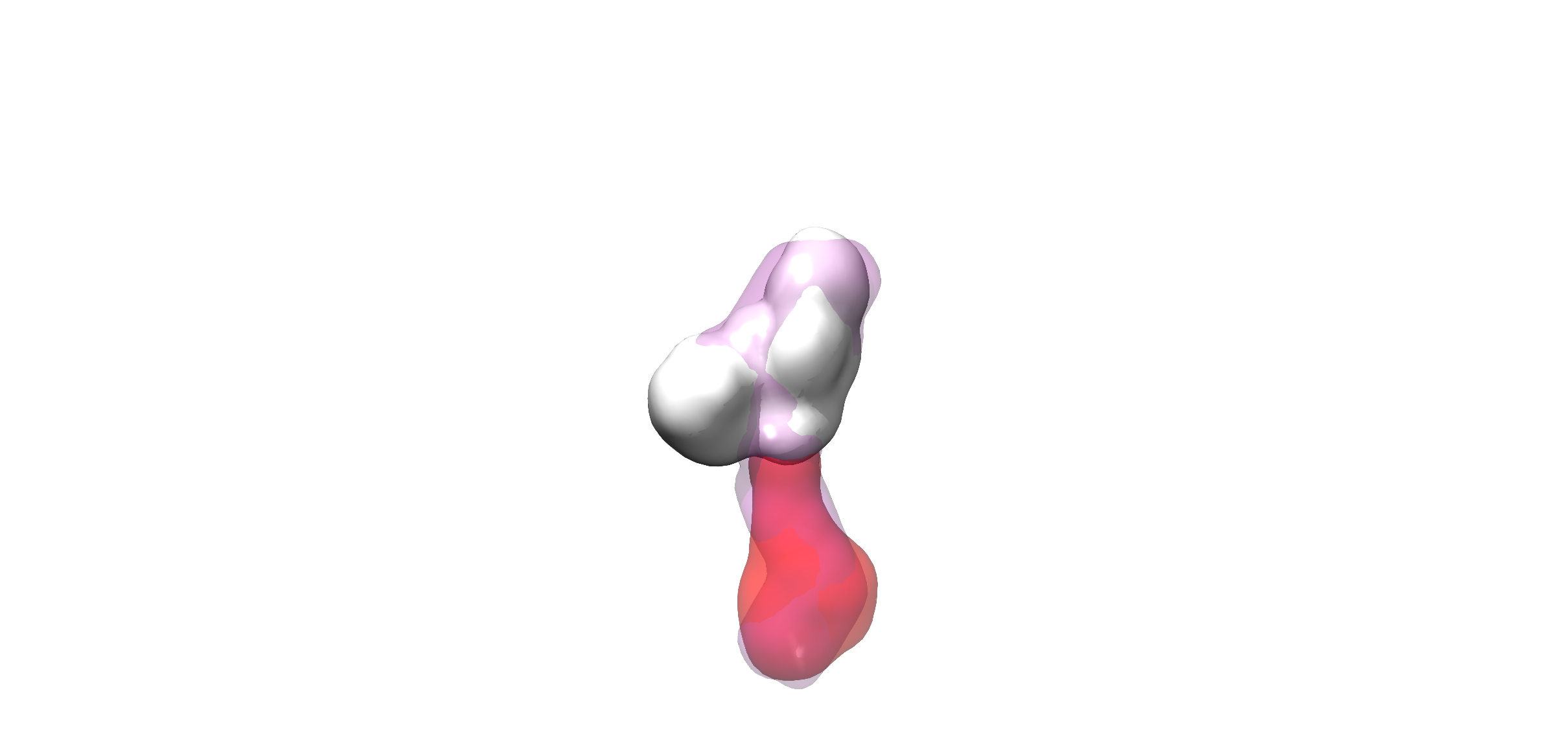

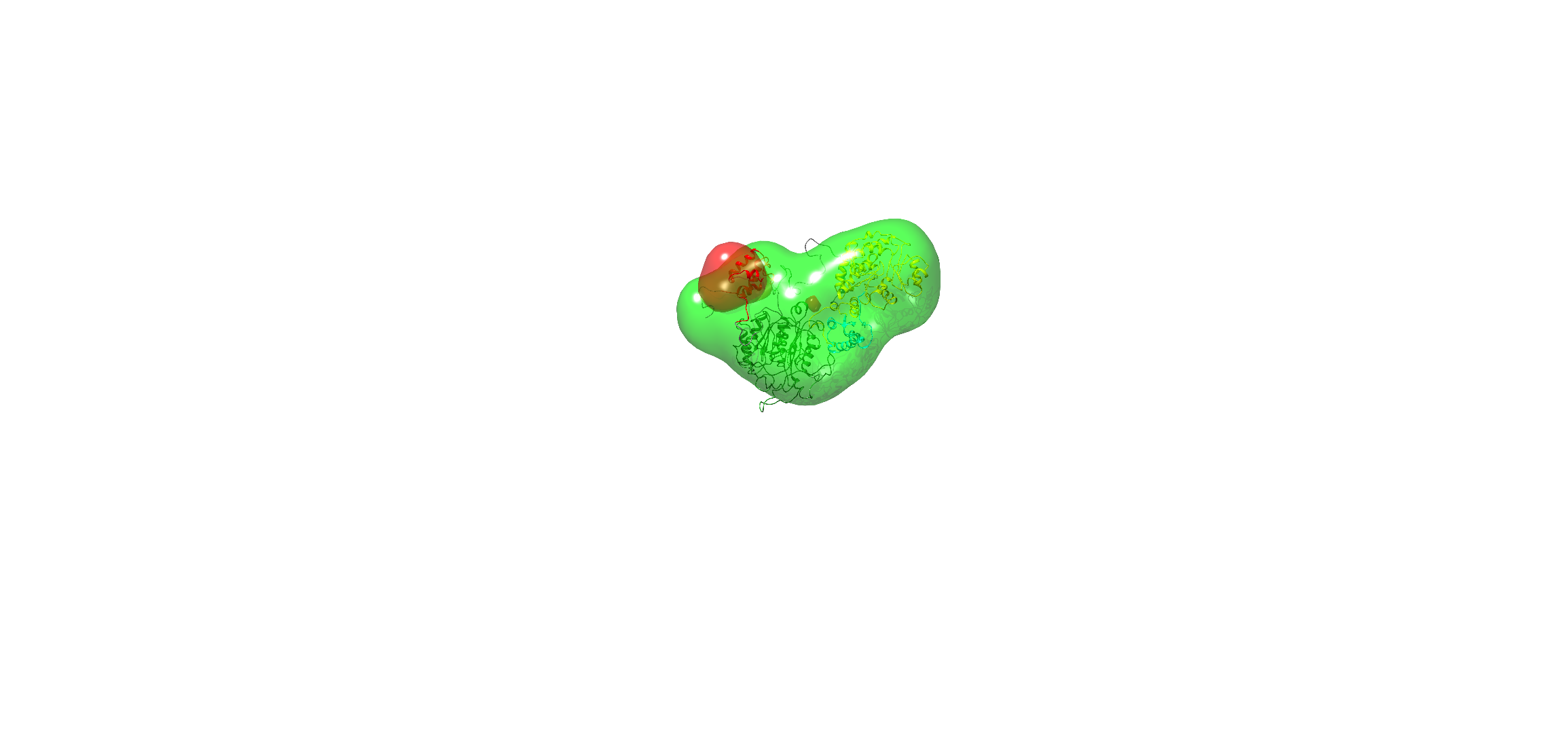

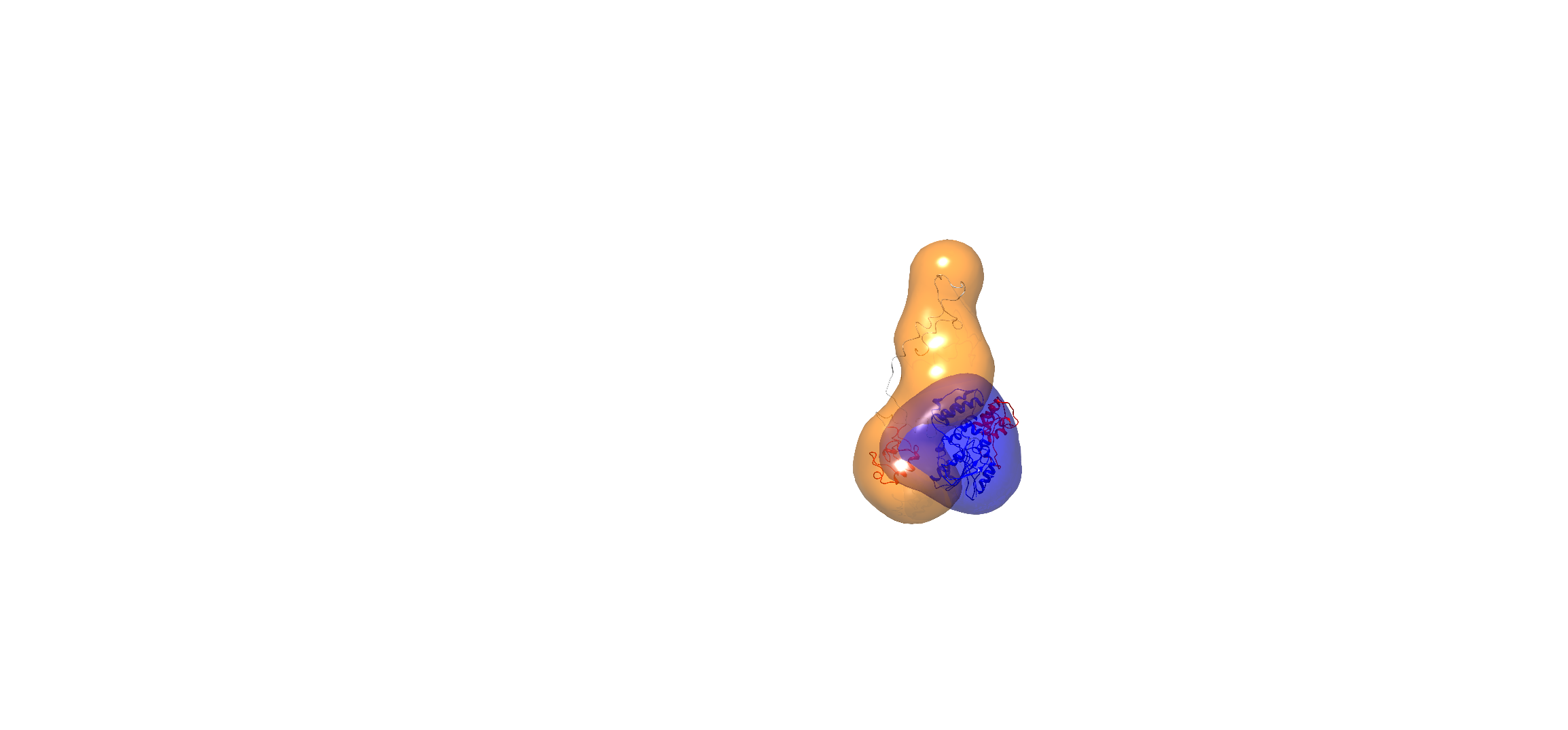


**a**

**b**

**Fig. S6.** Comparison of the Pks13, fACP1-KS-AT and fACP2-TE envelopes. (a) Superposition of the GASBOR envelopes of Pks13 (pink), fACP1-KS-AT (grey) and fACP2-TE (red). (b) Superposition of the MONSA low-resolution structures with the hybrid models of fACP1-KS-AT and fACP2-TE. The region attributed by MONSA to ACP1, KS-AT, TE and to the region 1062-1444 (post-AT linker until the TE domain) in fACP2-TE are respectively in red, green, dark blue and orange. Superposed are the HR models of ACPs (red), KS (dark green), LD (cyan), AT (yellow) and TE (dark blue).

**Fig. S7.** Impact of ionic strength on Pks13 activity. Influence of the NaCl concentration on the Pks13 condensation activity. Assays were performed in the presence of carboxypalmitoyl-CoA, [1-^14^C] lauric acid, FadD32 (for activation of the fatty acid into acyl-AMP) and phosphopantetheinylated Pks13, and incubated for 6 h at 30 °C in the appropriate reaction medium (see Experimental procedures). After chemical treatment and extraction, the reaction products were quantified by phosphorimaging. The relative enzymatic activity was fixed to 100% at 23 mM NaCl.

**a**


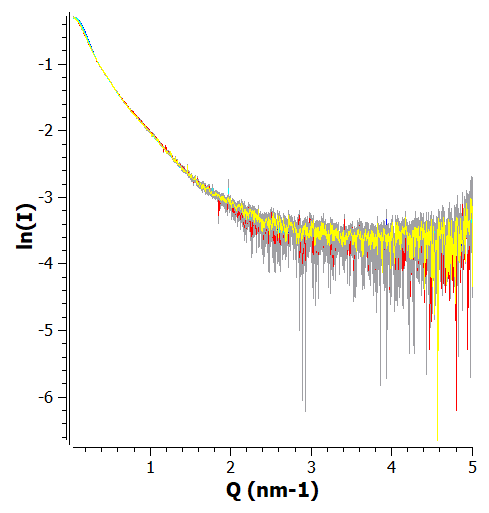

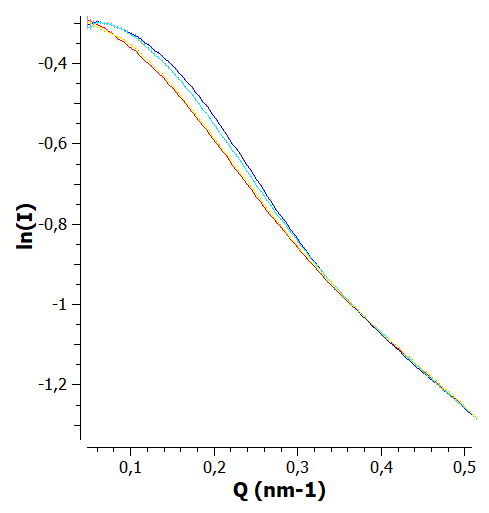


**b**


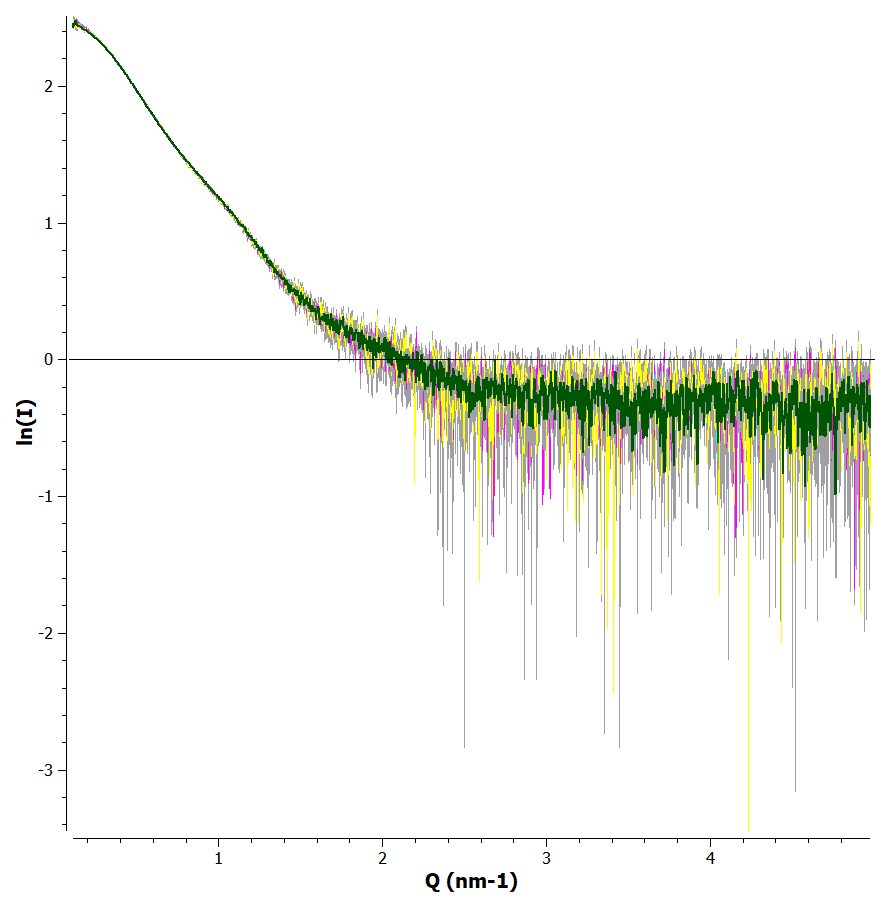

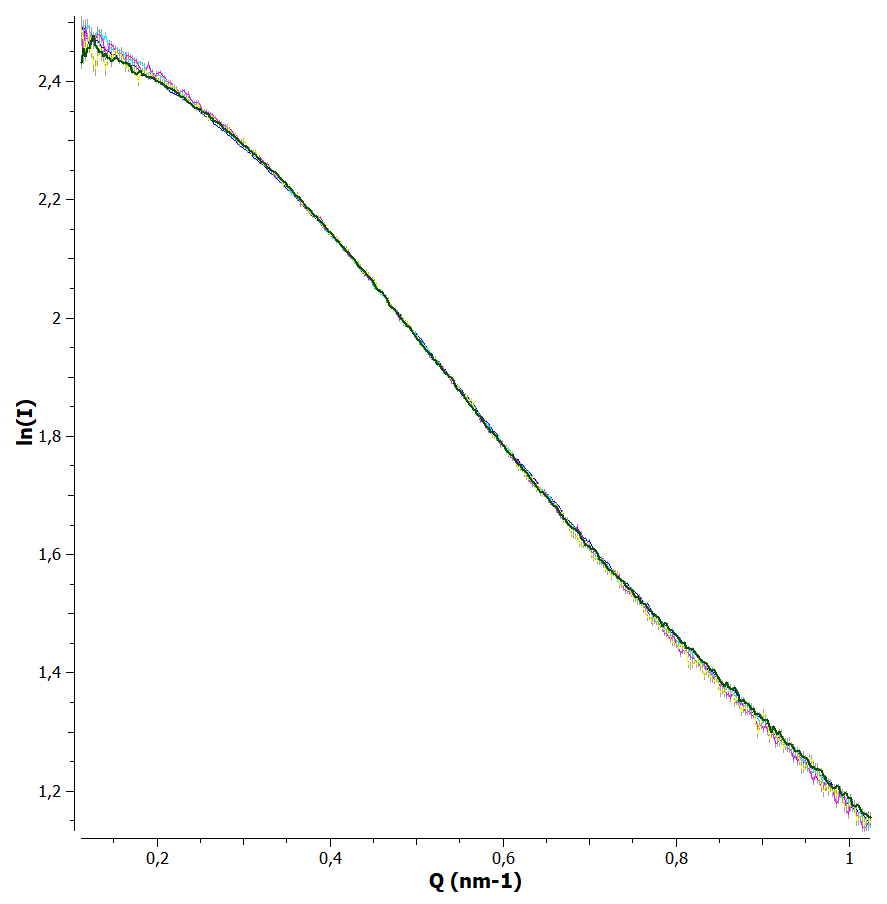


**c**


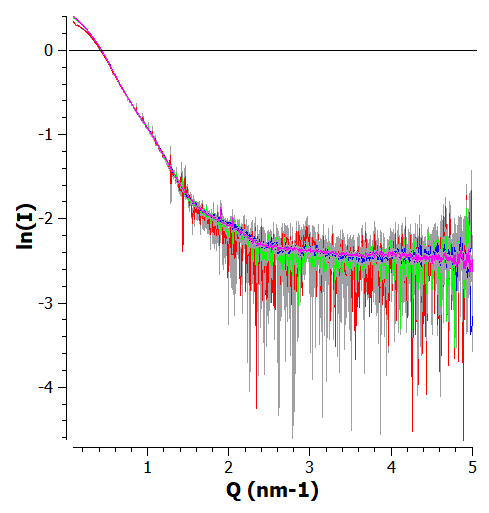

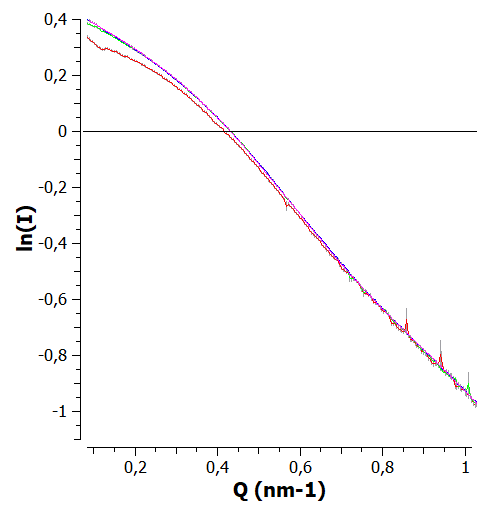


**Fig. S8.** Analysis of concentration dependence for SAXS data collected in batch mode (part 1/2).

**d**


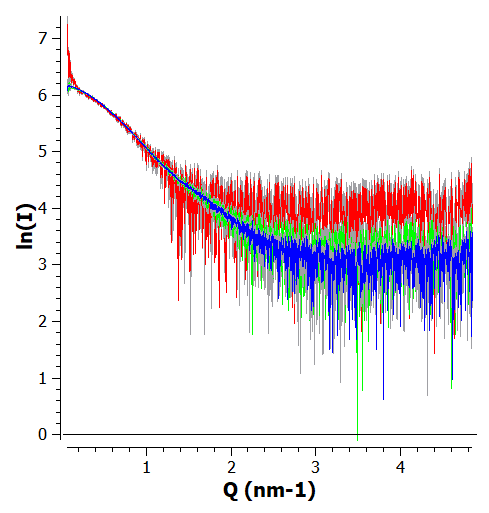

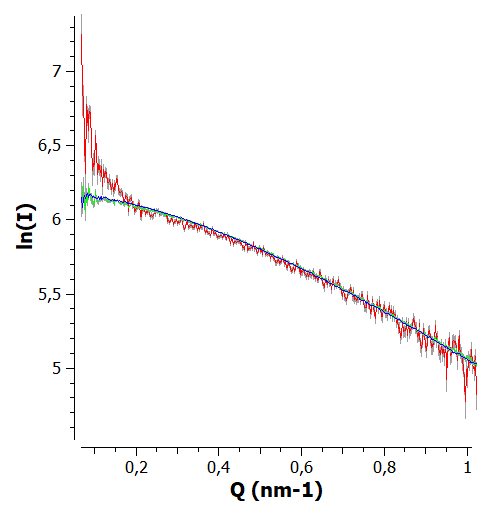


**e**


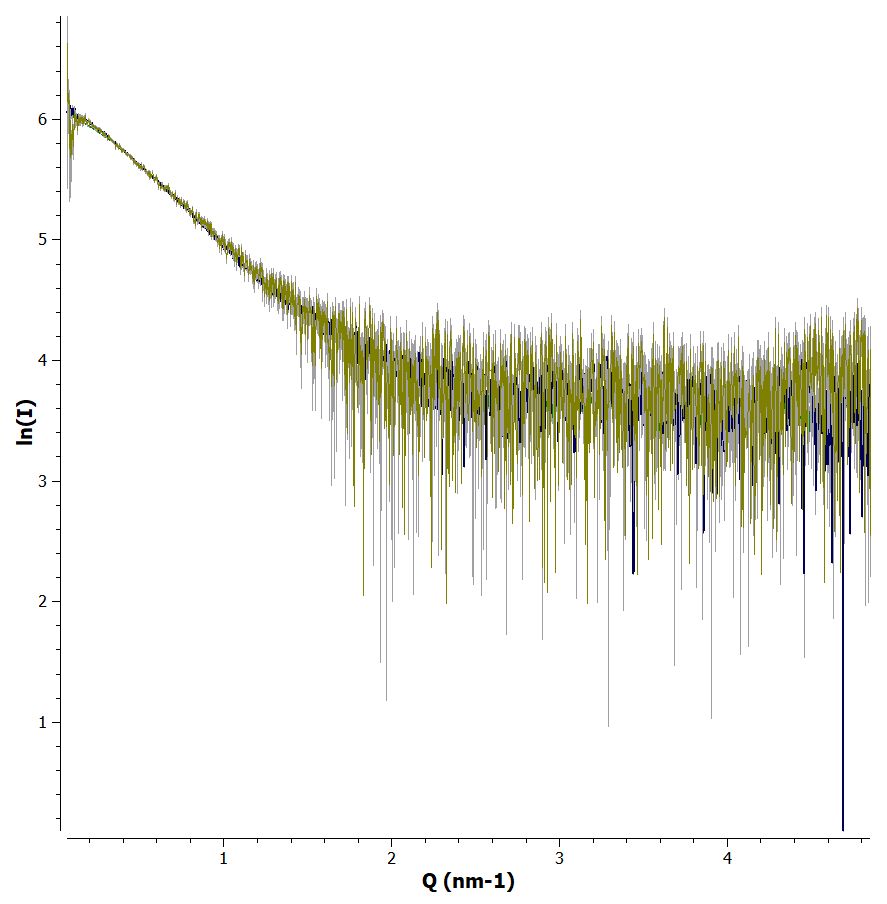

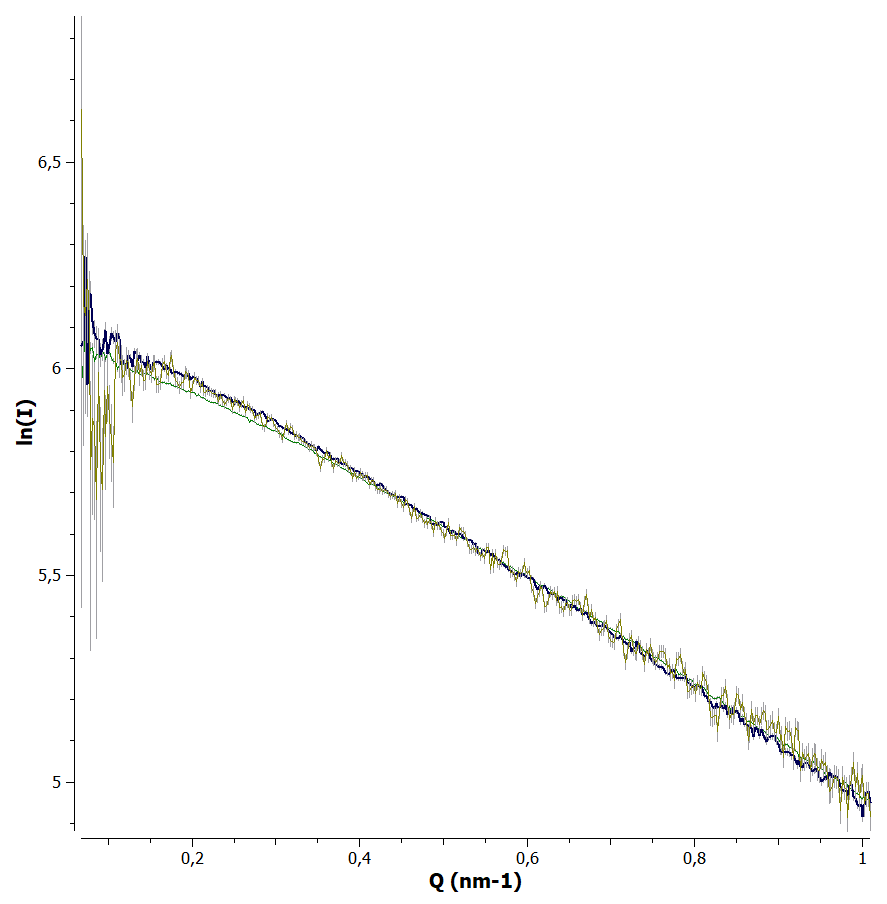


**f**


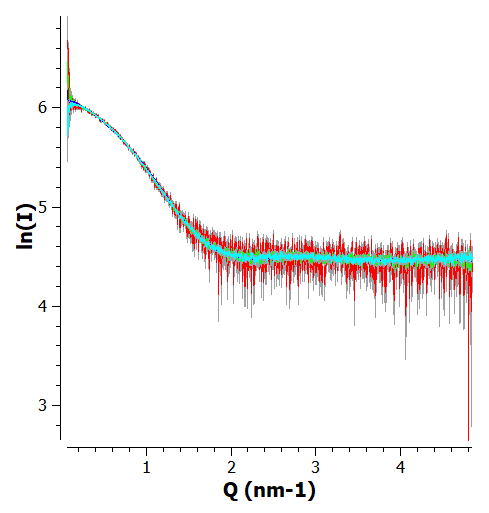

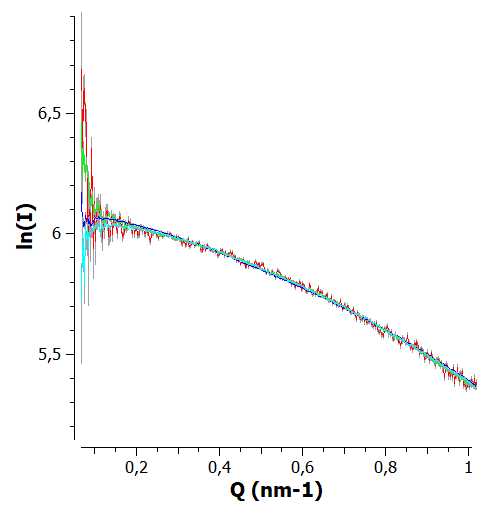


**Fig. S8.** Analysis of concentration dependence for SAXS data collected in batch mode (part 2/2). Left, SAXS intensity normalized by concentration. Right, zoom at low *Q*-value. (a) Pks13: red, apo-form at 2.0 mg/ml; yellow, holo-form at 2.0 mg/ml; light blue, apo-form at 5.2 mg/ml; dark blue, apo-form at 10.4 mg/ml. Data used in this study are a merge between the data at 2.0 and 10.4 mg/ml. (b) fACP1-KS-AT: yellow 3.2 mg/ml at 300 mM NaCl; pink, 3.7 mg/ml at 300 mM NaCl; light blue, 5.8 mg/ml at 300 mM NaCl; dark blue, 8 mg/ml at 300 mM NaCl; dark green, 5.7 mg/ml at 50 mM NaCl (data used in this study). (c) fKS-AT: red: 1.2 m mg/ml; light green, 1.9 mg/ml; dark blue, 5.2 mg/ml, pink, 11.0 mg/ml. Data used in this study are a merge between 1.2 and 11 mg/ml. (d) AT52: red, 0.7 mg/ml; light green, 4.0 mg/ml; dark blue, 9.6 mg/ml (data used in this study). (e) fACP2-TE: light green, 1 mg/ml; dark green, 5 mg/ml (data used in this study); dark blue, 12.6 mg/ml. B: zoom at low Q-value. (f) fTE: red, 1.0 mg/ml; light green, 4.1 mg/ml; dark blue, 18.2 mg/ml; light blue, 10.3 mg/ml (data used in this study).

| Pks13 | 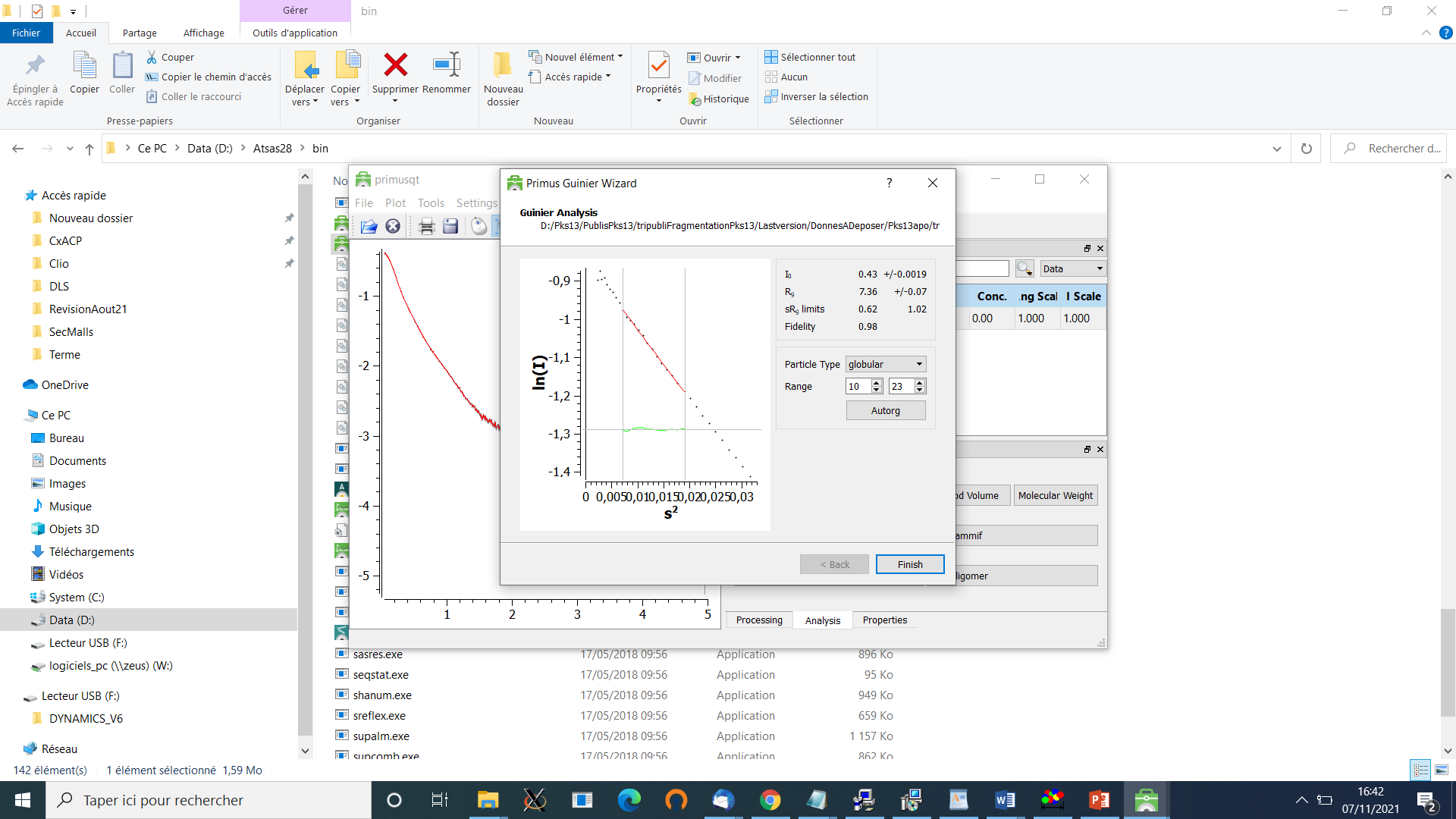Limit *Q*.*R*_g_=1  Fidelity 98% | 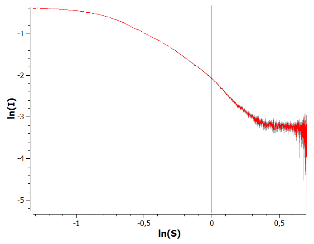 | Holo-Pks13 | 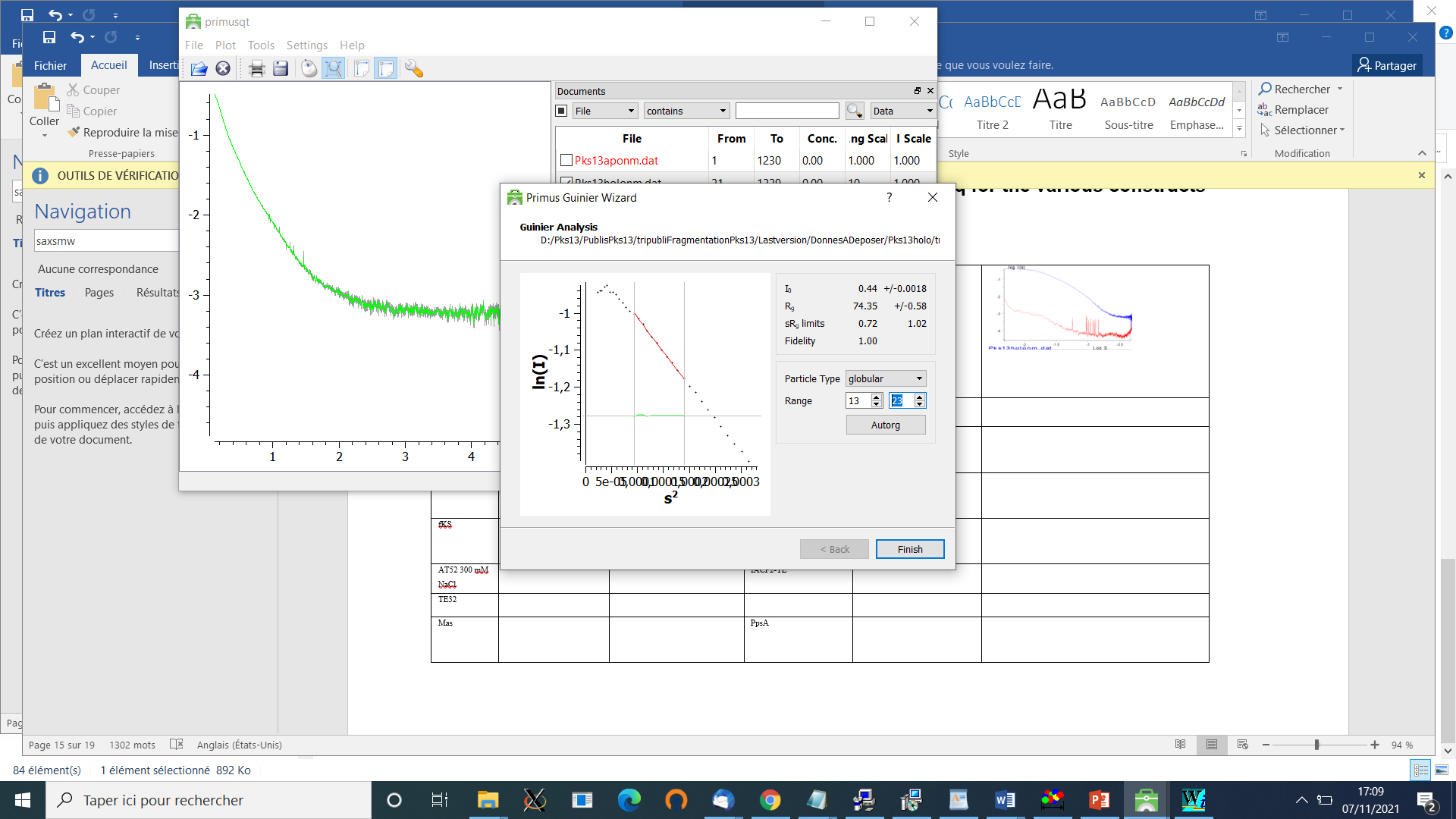Limit *Q*.*R*_g_=1  Fidelity 100% | 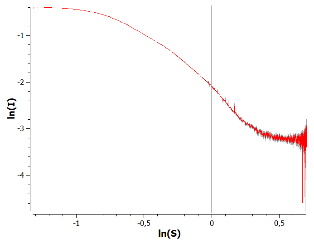 |
| --- | --- | --- | --- | --- | --- |
| Pks13(S1533A) | 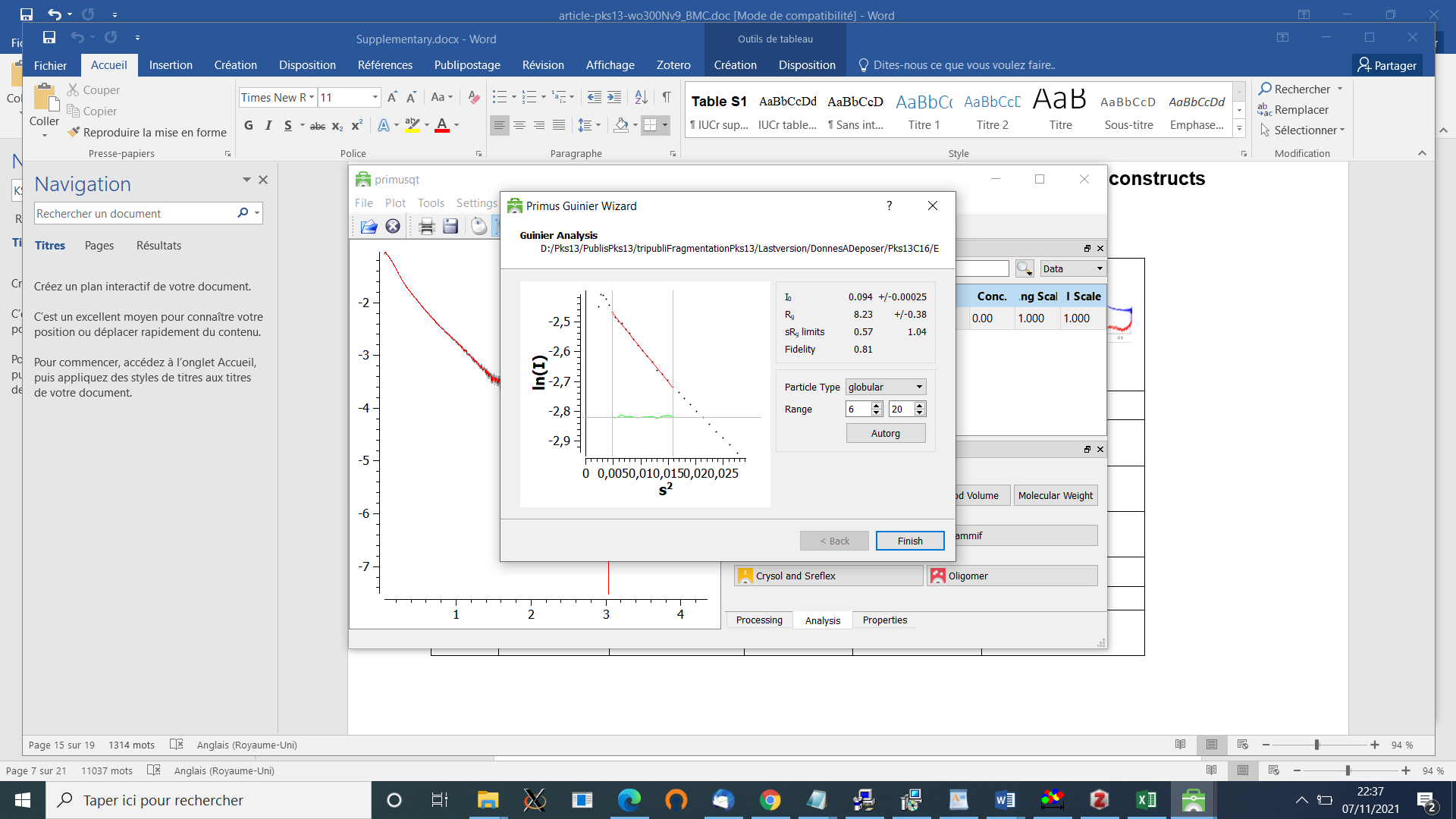 Limit *Q*.*R*_g_=1  Fidelity 81% | 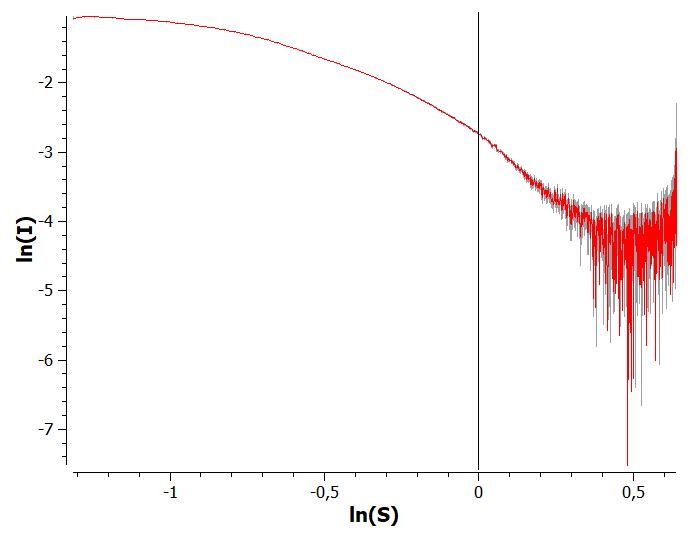 | Pks13(S1533A)  in HEPES | 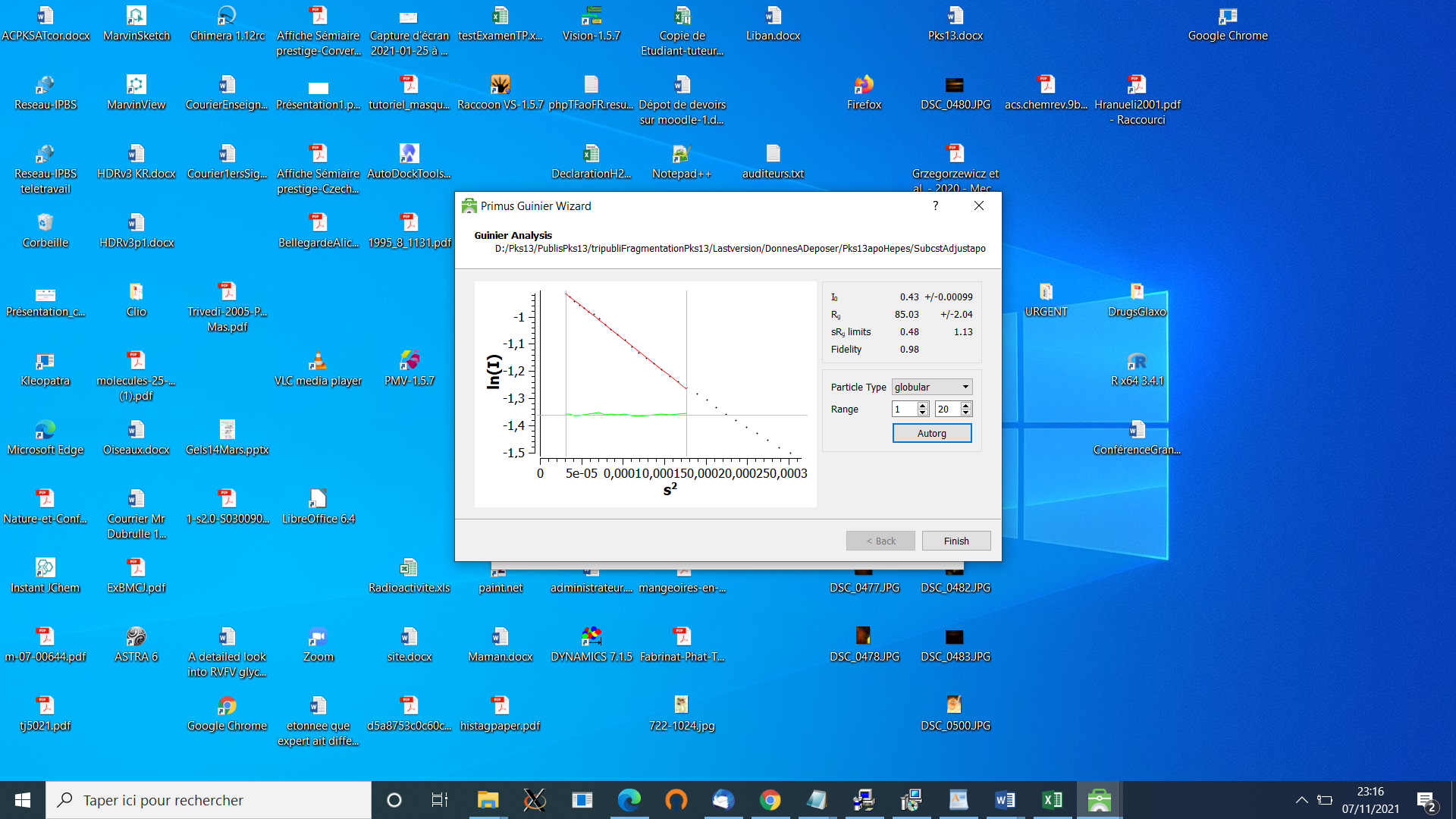Limit *Q*.*R*_g_=1  Fidelity 98% | 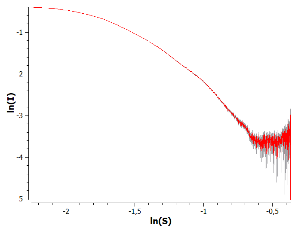 |
| C16-Pks13(S1533A)  Monomer | 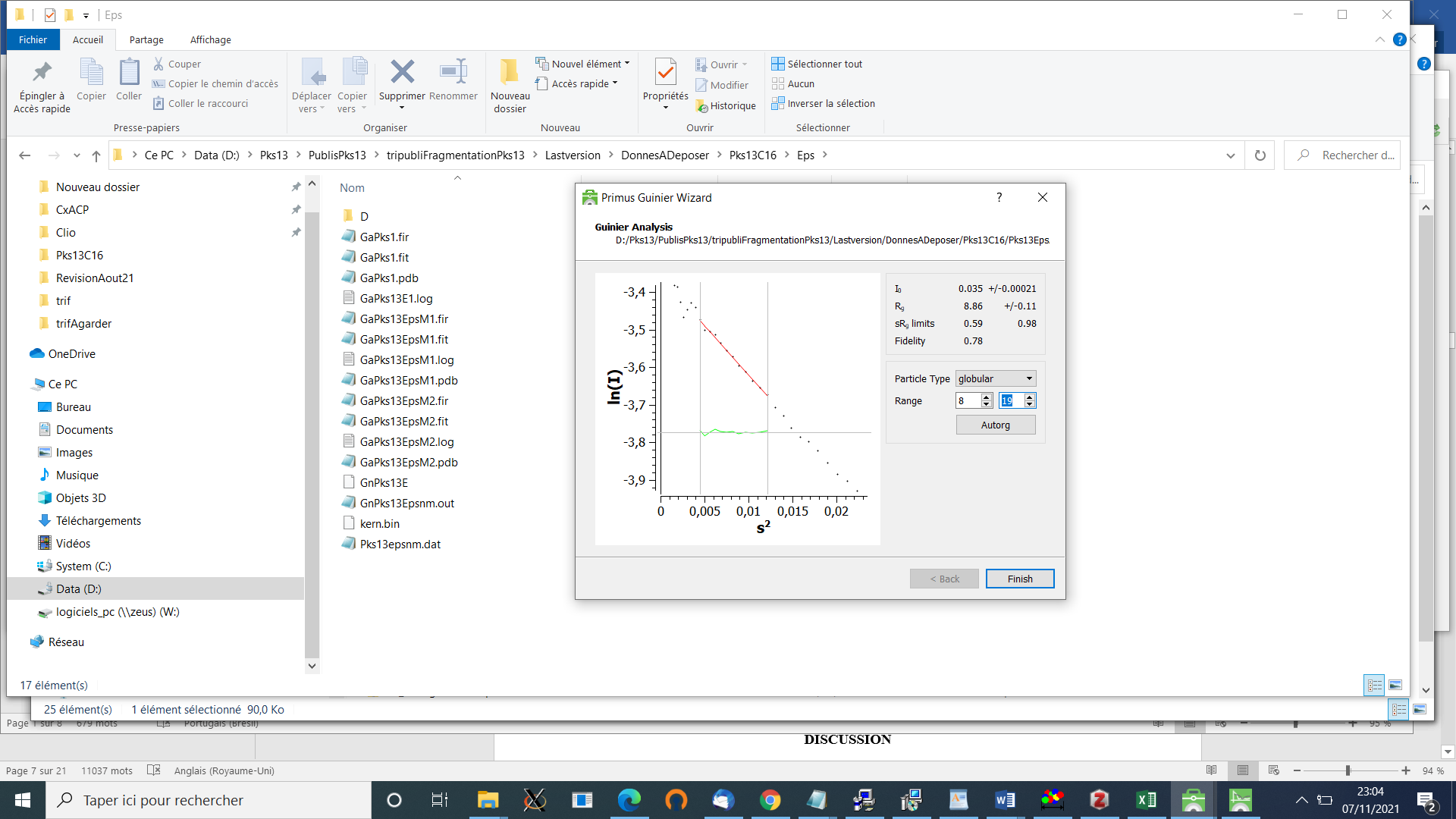Limit *Q*.*R*_g_=1  Fidelity 78% | 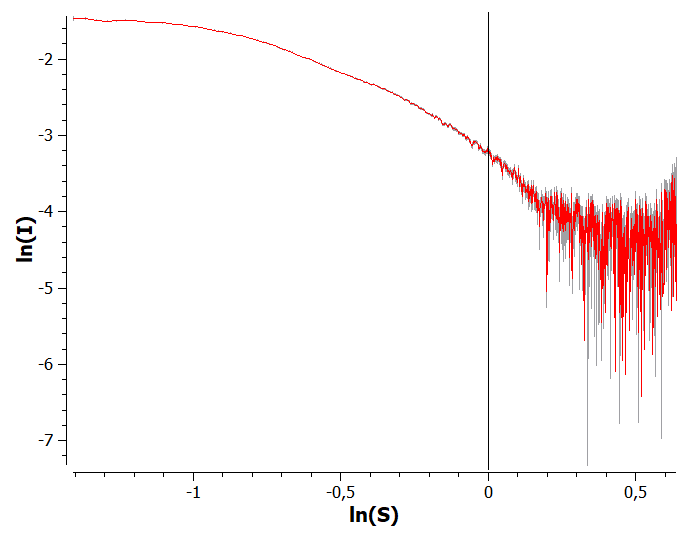 | C16-Pks13(S1533A)  Dimer | 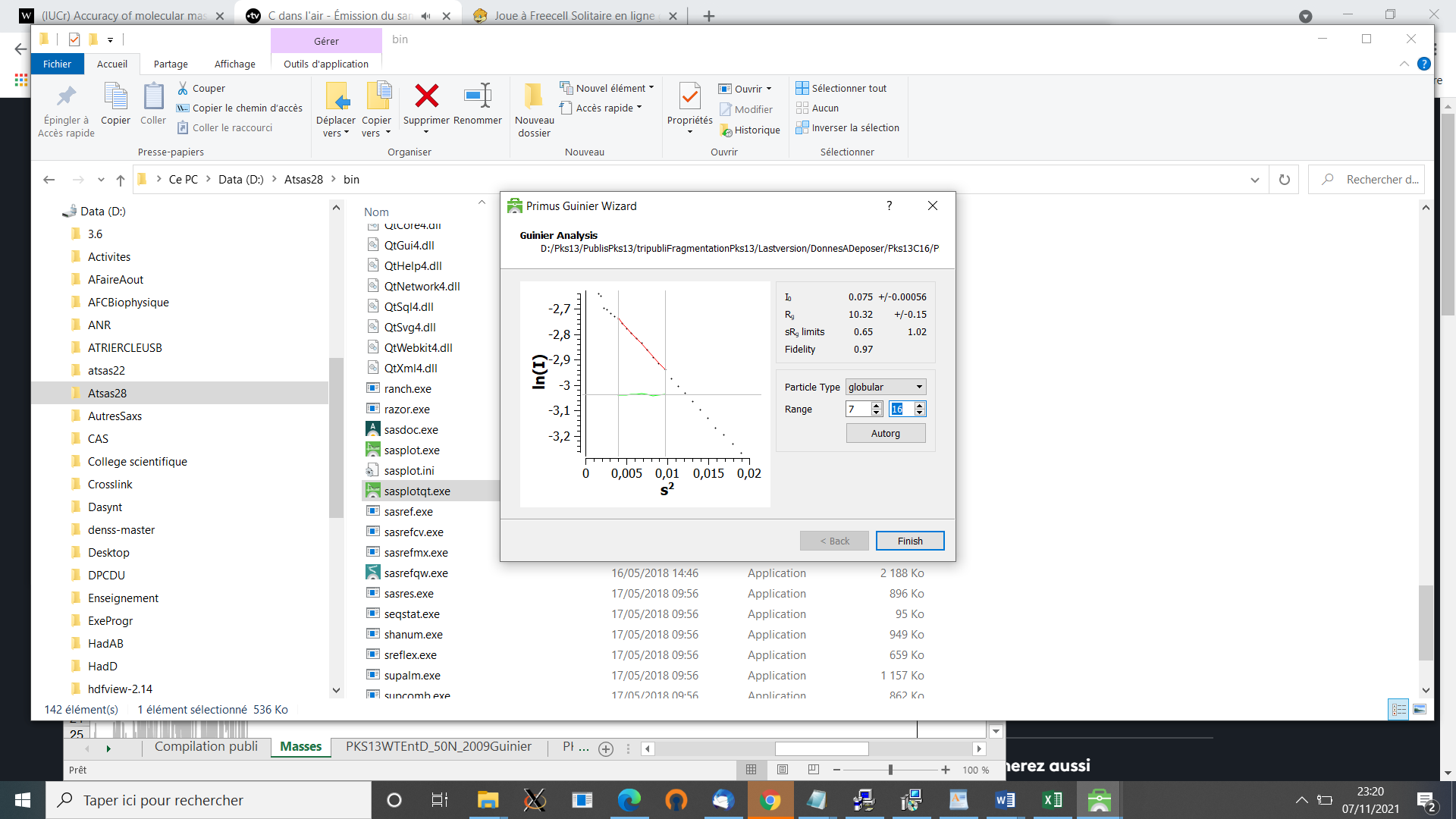Limit *Q*.*R*_g_=1  Fidelity 97% | 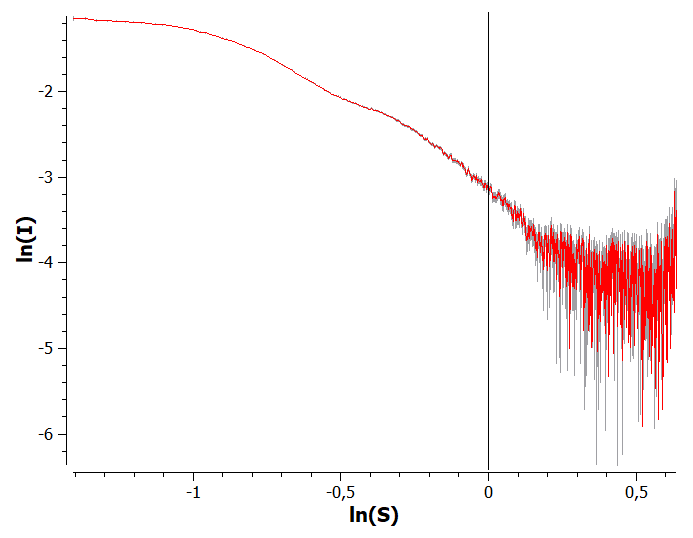 |

**Fig. S9.** Guinier analysis and plot of ln I(s) versus ln(s) for the various Pks13 constructs (part 1/3). Here s=*Q*=4πsin(θ)/λ in nm^-1^.

| fACP1‑KS‑AT | 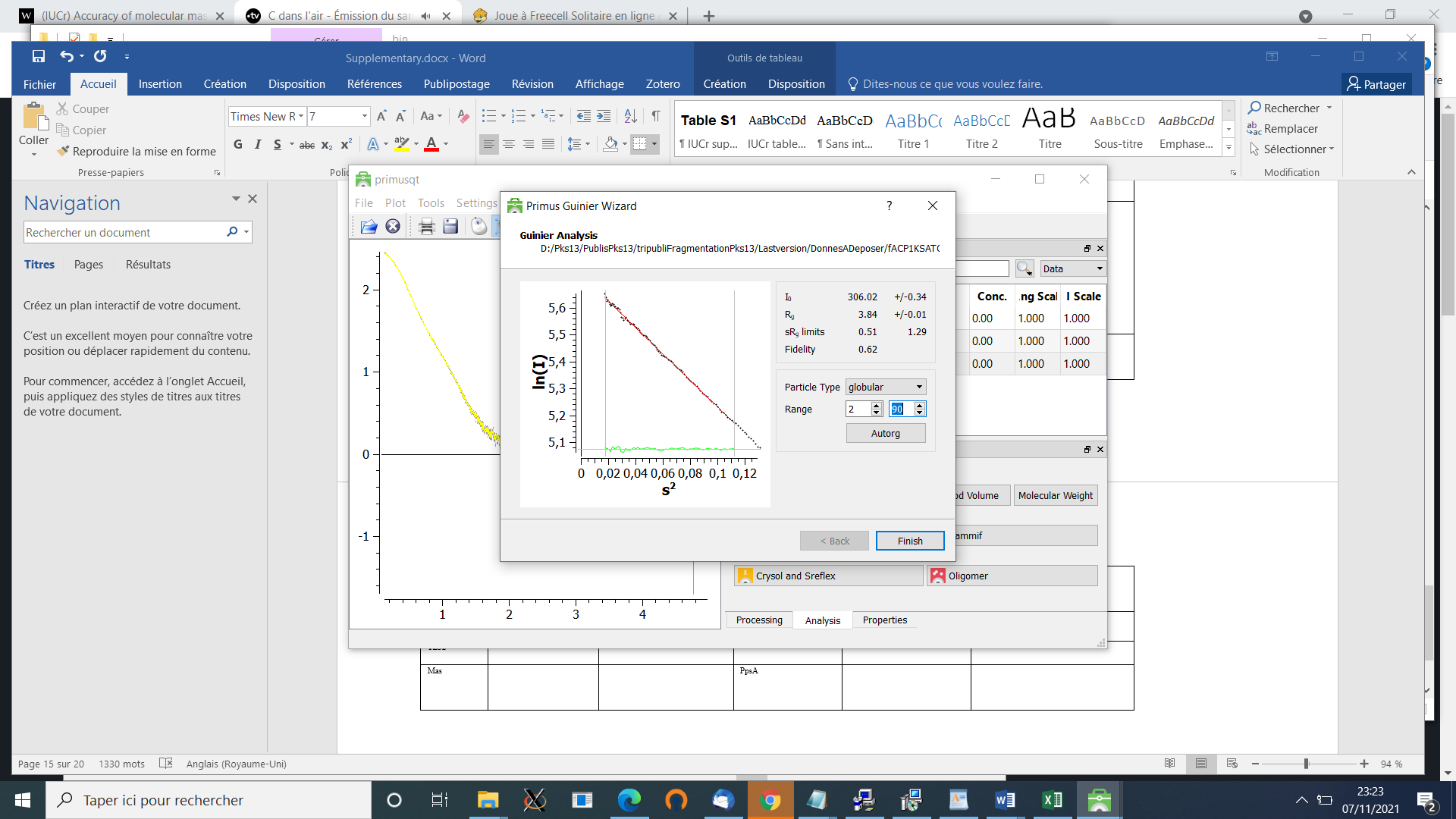 Limit *Q*.*R*_g_=1.3  Fidelity 100% | 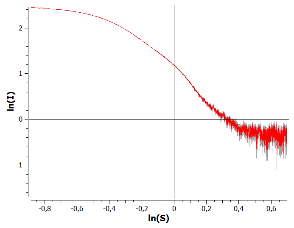 | fKS‑AT | 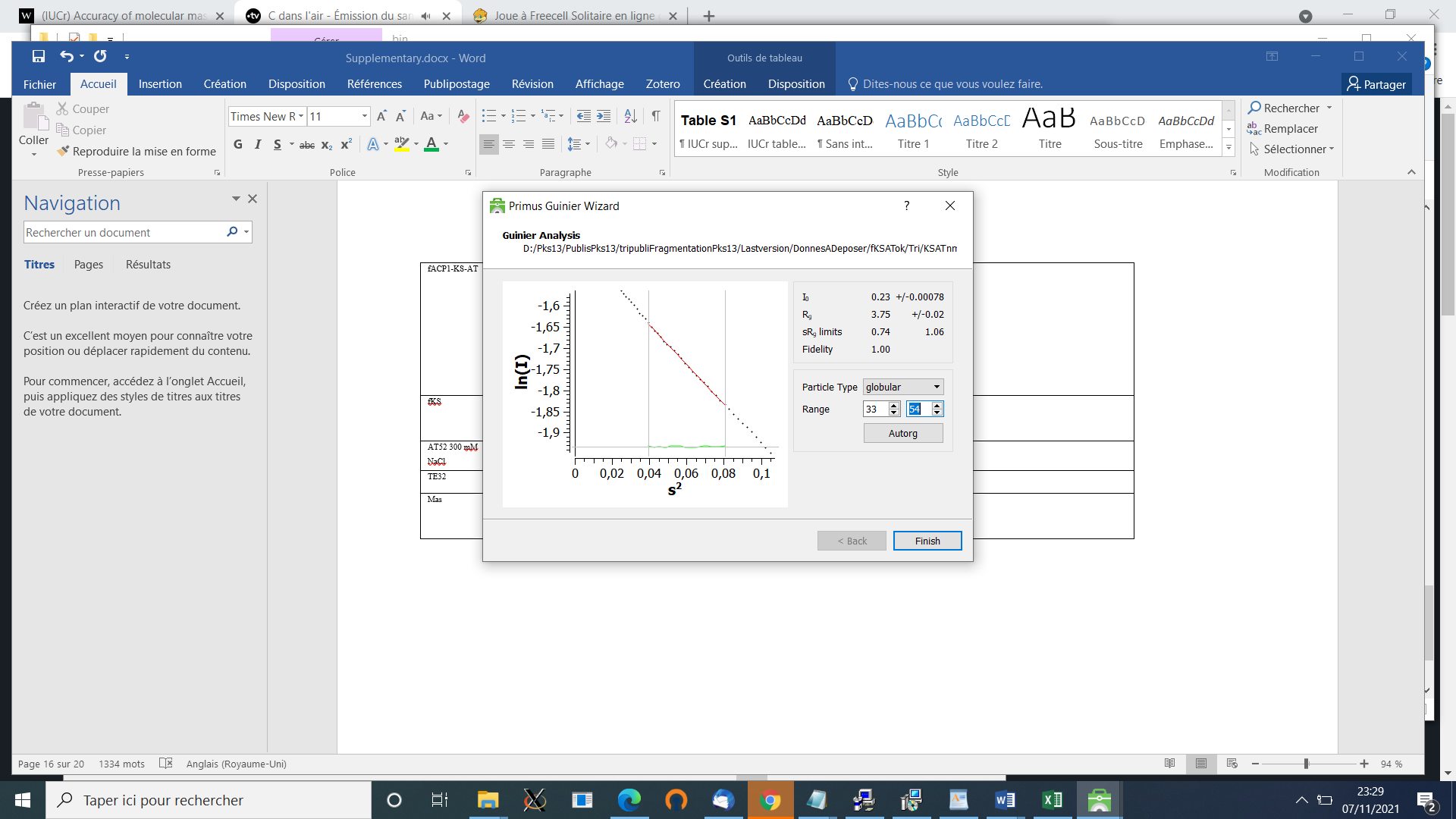Limit *Q*.*R*_g_=1  Fidelity 100% | 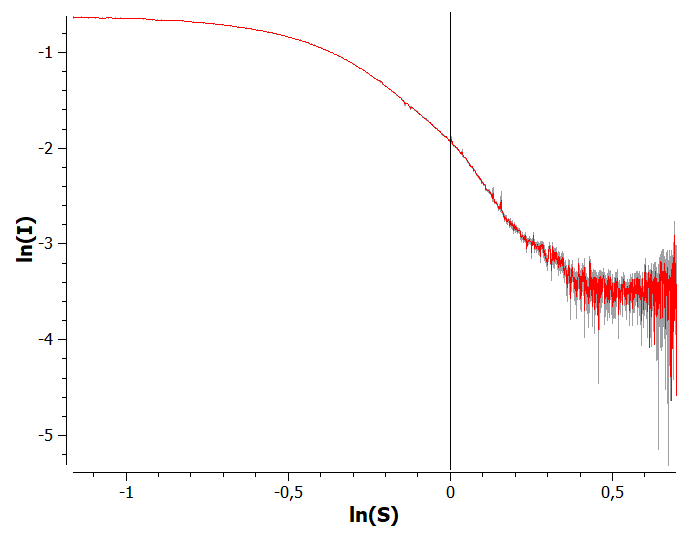 |
| --- | --- | --- | --- | --- | --- |
| fKS | 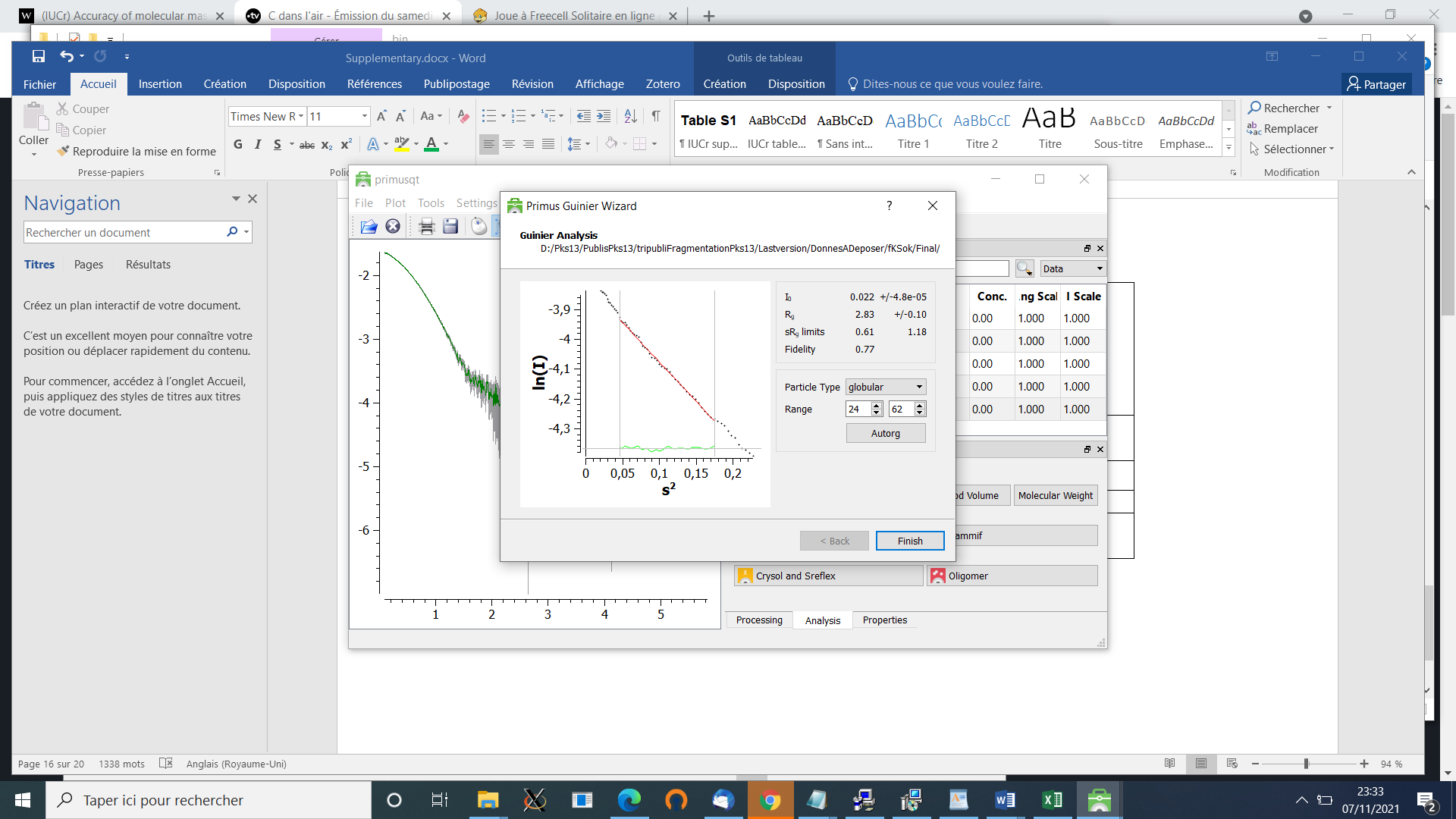Limit *Q*.*R*_g_=1.3  Fidelity 77% | 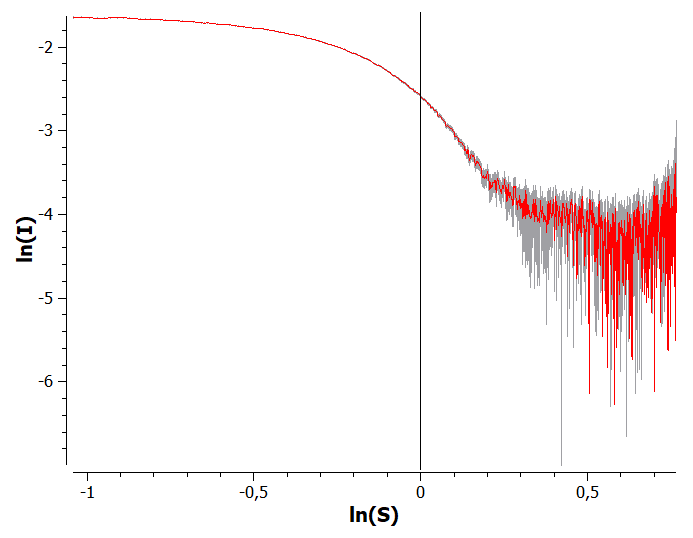 | fAT | 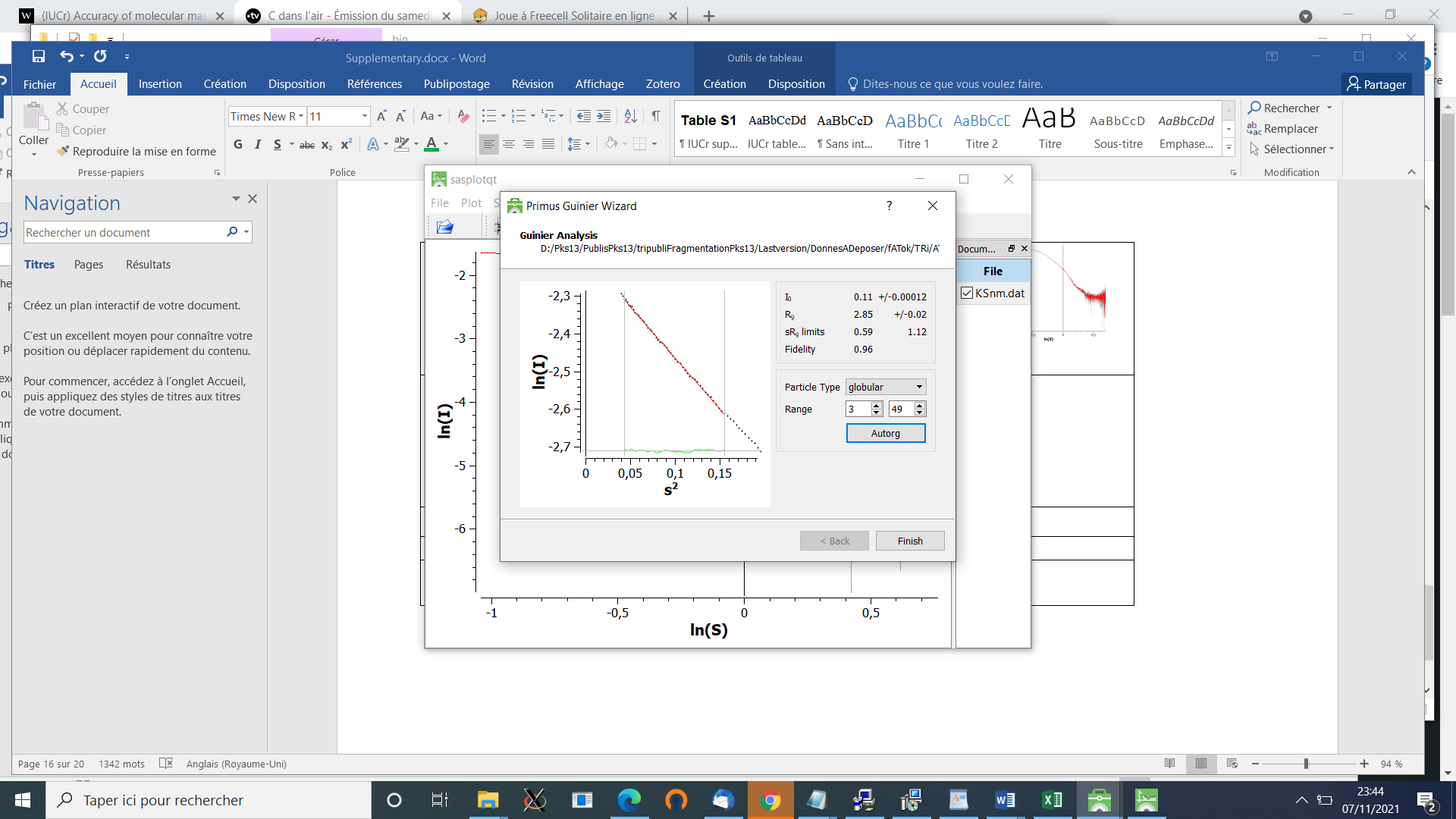Limit *Q*.*R*_g_=1.3  Fidelity 96% | 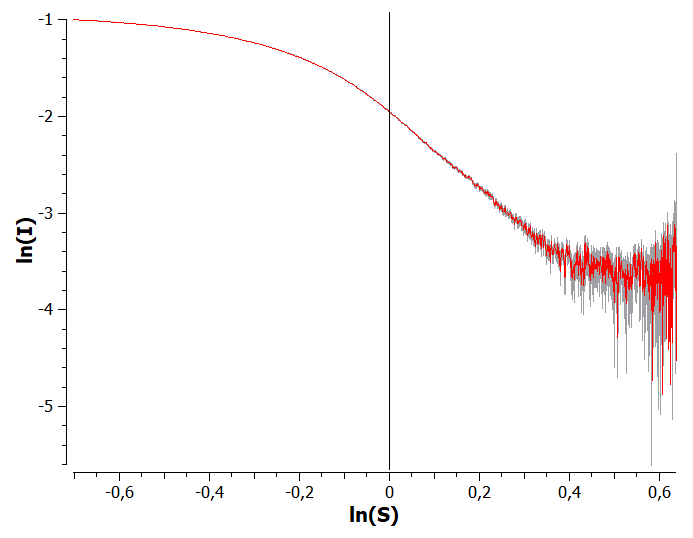 |
| AT52 | 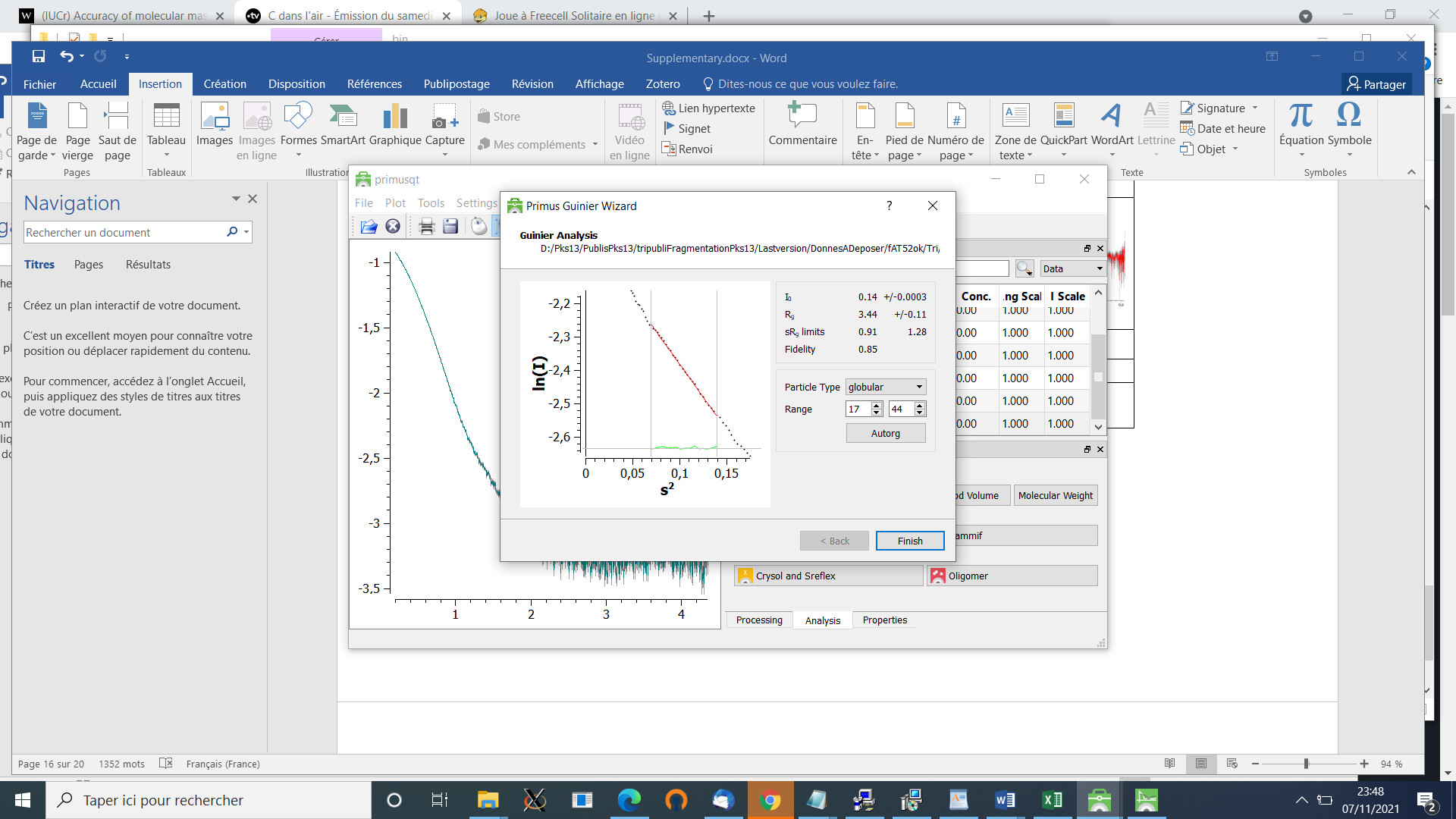Limit *Q*.*R*_g_=1.3  Fidelity 85% | 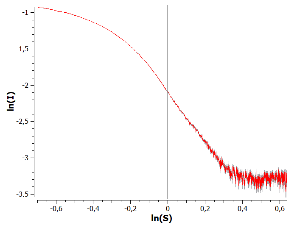 | fACP2-TE | 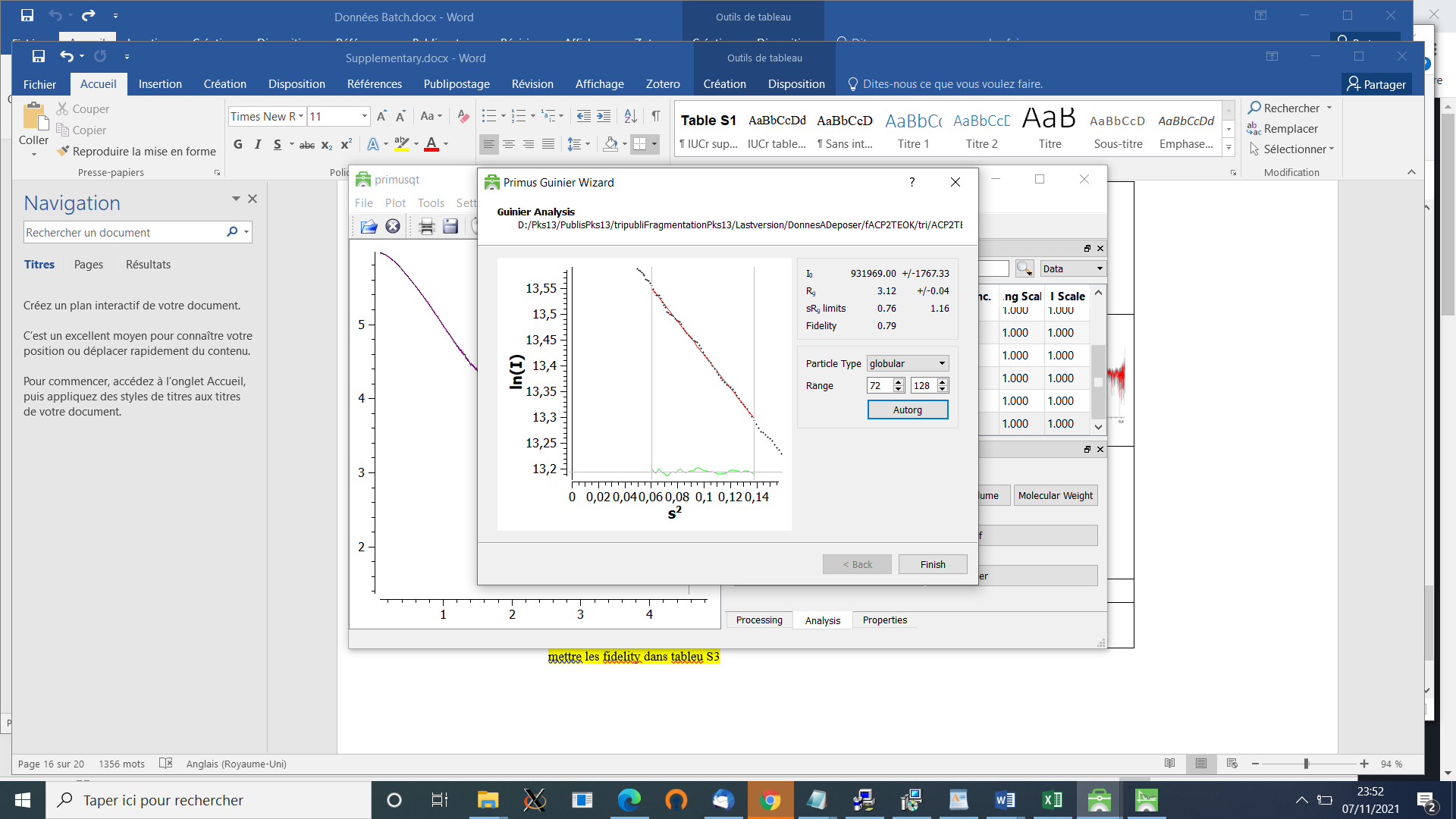Limit *Q*.*R*_g_=1.1  Fidelity 79% | 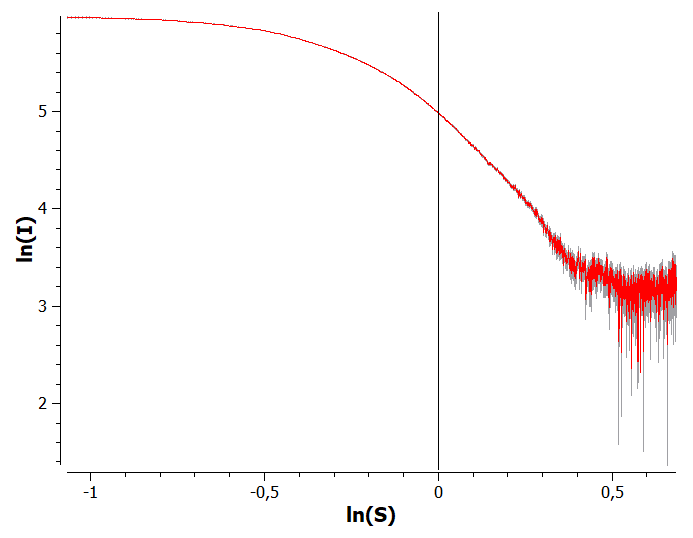 |

**Fig. S9.** Guinier analysis and plot of ln I(s) versus ln(s) for the various Pks13 constructs (part 2/3). Here s=*Q*=4πsin(θ)/λ in nm^-1^.

| fTE | 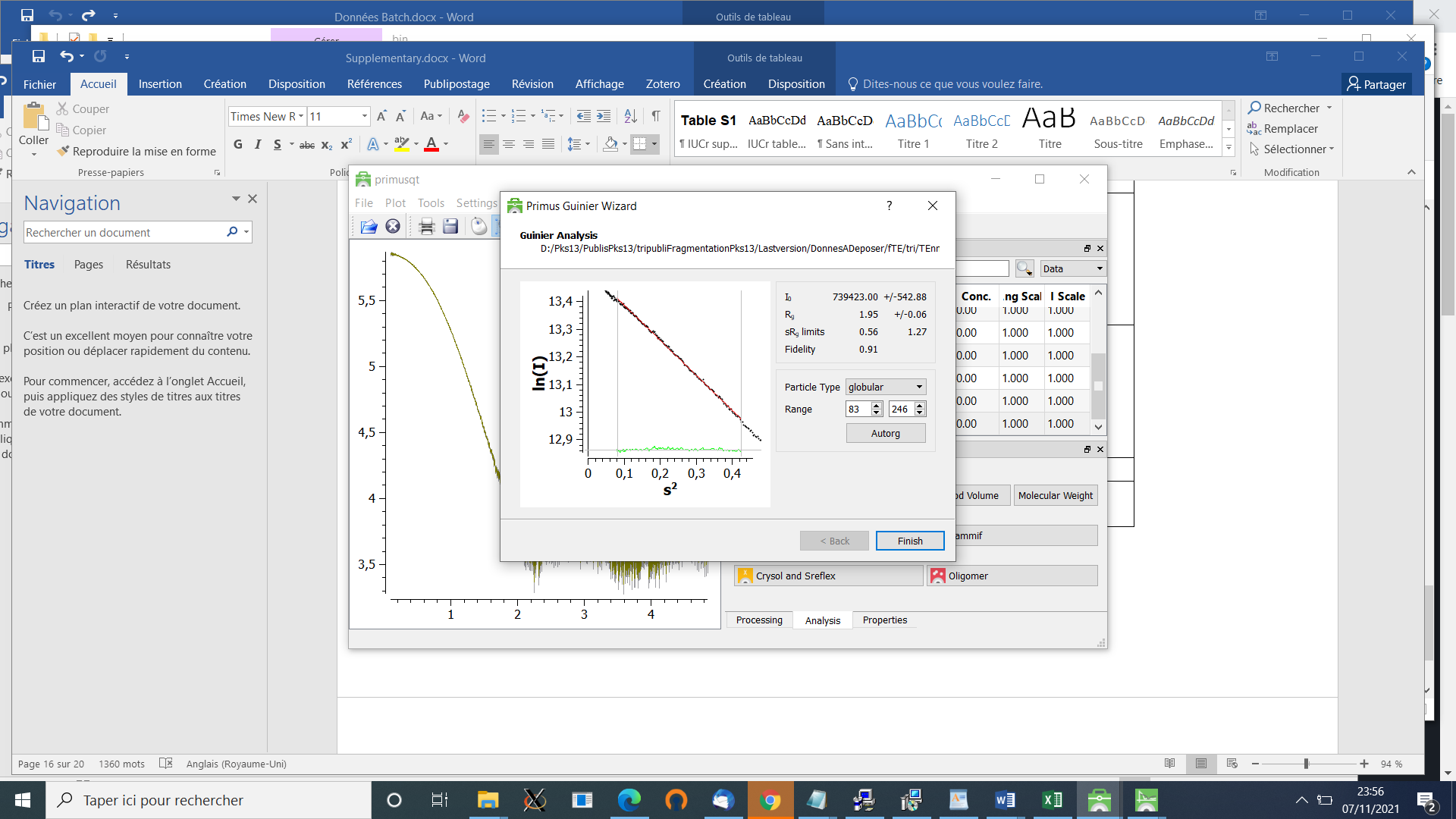Limit *Q*.*R*_g_=1.3  Fidelity 91% | 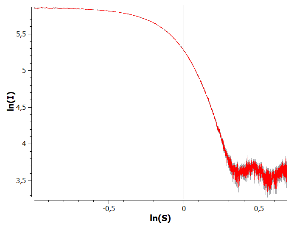 |  |  |  |
| --- | --- | --- | --- | --- | --- |
| Mas | 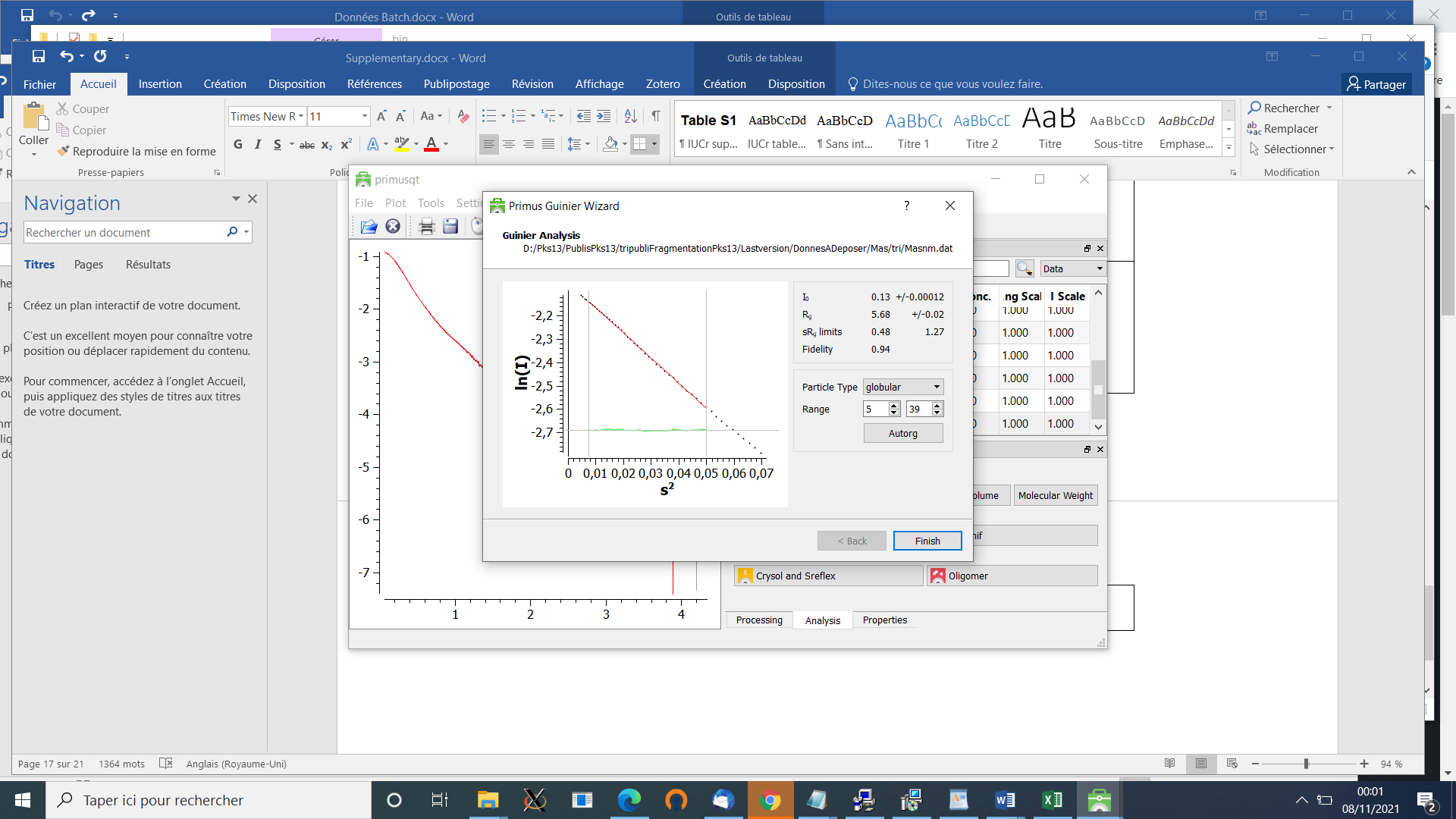  Limit *Q*.*R*_g_=1.3  Fidelity 94% | 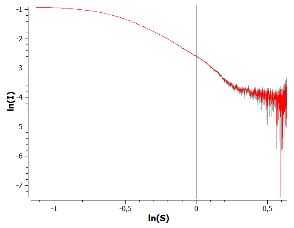 | PpsA | 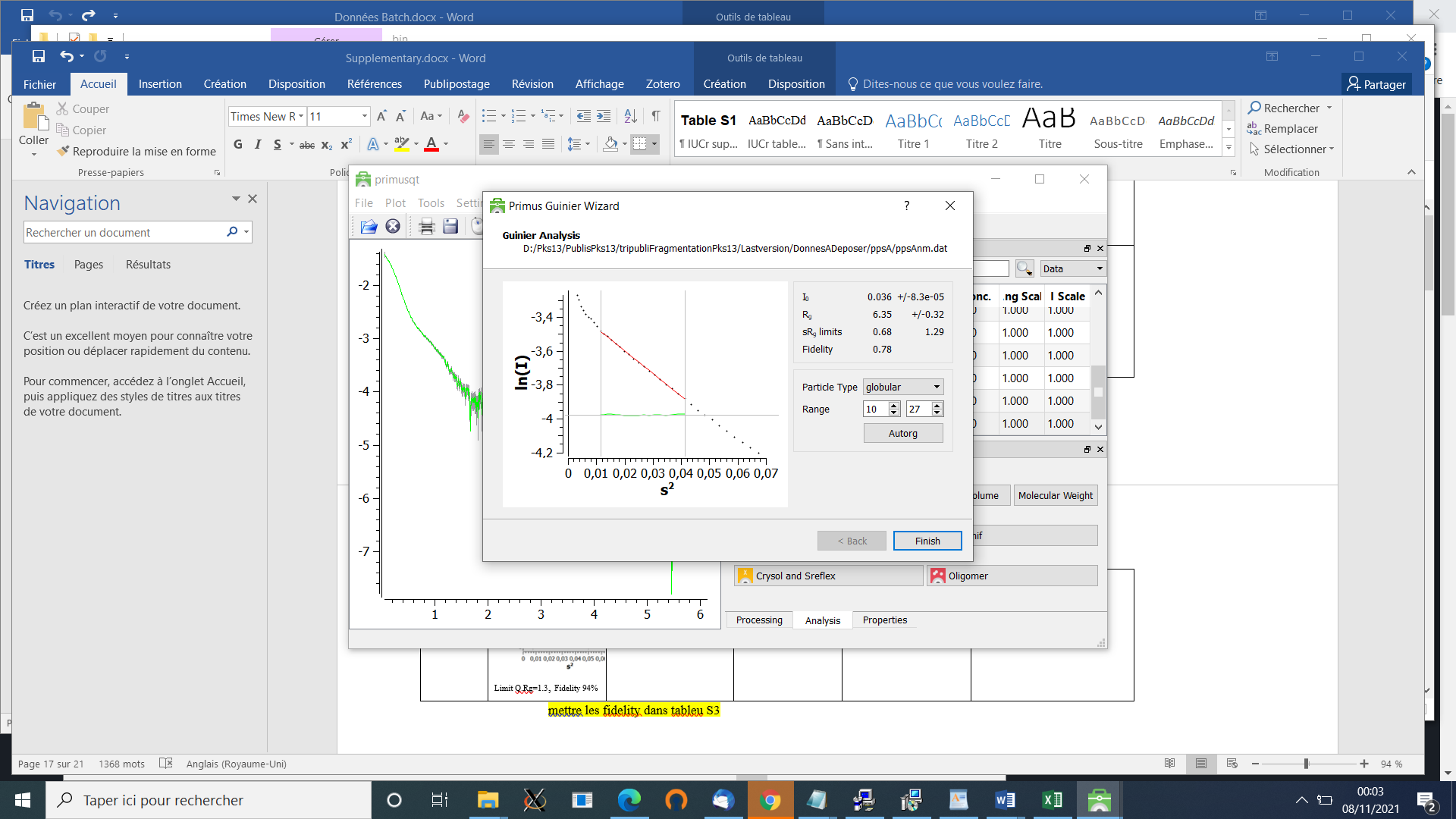  Limit *Q*.*R*_g_=1.3  Fidelity 78% | 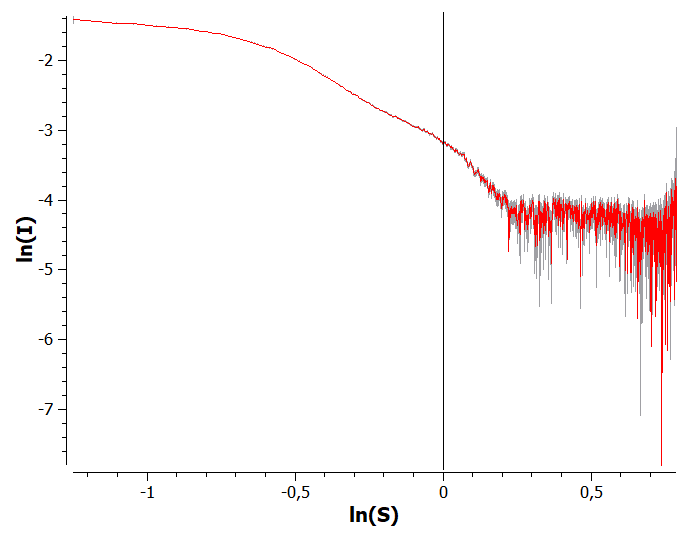 |

**Fig. S9.** Guinier analysis and plot of ln I(s) versus ln(s) for the various Pks13 constructs (part 3/3). Here s=*Q*=4πsin(θ)/λ in nm^-1^.
